# Construction of a Plasmid-Free *Escherichia coli* Strain with Enhanced Heme Supply to Produce Active Hemoglobins

**DOI:** 10.3390/metabo15030151

**Published:** 2025-02-23

**Authors:** Zihan Zhang, Baodong Hu, Jingwen Zhou, Jianghua Li, Jian Chen, Guocheng Du, Xinrui Zhao

**Affiliations:** 1 Science Center for Future Foods, Jiangnan University, 1800 Lihu Road, Wuxi 214122, China; 6220201061@stu.jiangnan.edu.cn (Z.Z.); 8202306026@jiangnan.edu.cn (B.H.); zhoujw1982@jiangnan.edu.cn (J.Z.); lijianghua@jiangnan.edu.cn (J.L.); jchen@jiangnan.edu.cn (J.C.); 2 Key Laboratory of Industrial Biotechnology, Ministry of Education, School of Biotechnology, Jiangnan University, 1800 Lihu Road, Wuxi 214122, China; 3 Key Laboratory of Carbohydrate Chemistry and Biotechnology, Ministry of Education, Jiangnan University, 1800 Lihu Road, Wuxi 214122, China

**Keywords:** heme, biosynthesis, hemoglobin, plasmid-free, rate-limiting steps

## Abstract

Background: Heme is an important cofactor and plays crucial roles in the correct folding of hemoproteins. The synthesis of heme can be enhanced by the plasmid-based expression of heme biosynthetic genes. However, plasmid-based expression is genetically unstable and requires the utilization of antibiotics to maintain high copy numbers of plasmids. Methods: The rate-limiting steps in heme biosynthesis were first analyzed based on previous studies and the accumulation of heme intermediates was achieved by adding heme precursor (5-aminolevulinic acid, ALA). Next, the intracellular accumulation of porphyrin was increased by deleting the porphyrin transporter TolC. Finally, the heme synthetic genes were modified by integrating the *hemA* and *hemL* genes into the *cheW* and *yciQ* locus, assembling the rate-limiting enzymes HemC and HemD with RIAD-RIDD tags, replacing the promoters of *hemE/hemH* genes with the constitutive promoter P_J23100_, and deleting the heme degradation gene *yfeX*. Results: An enhanced heme supply HEME2 strain was obtained with a heme titer of 0.14 mg/L, which was 4.60-fold higher than that of the C41(DE3) strain. The HEME2 strain was applied to produce human hemoglobin and leghemoglobin. The titer and peroxidase activity of human hemoglobin were 1.29-fold and 42.4% higher in the HEME2-hHb strain than the values in the control strain C41-hHb. In addition, the peroxidase activity and heme content of leghemoglobin were increased by 39.2% and 53.4% in the HEME2-sHb strain compared to the values in the control strain C41-sHb. Conclusions: A plasmid-free *Escherichia coli* C41(DE3) strain capable of efficient and stable heme supply was constructed and can be used for the production of high-active hemoglobins.

## 1. Introduction

Heme is a porphyrin derivative consisting of ferrous ion (Fe^2+^) and protoporphyrin [1]. It plays important roles in oxygen transport, electron transfer, and signal transduction [2], and it is widely used in healthcare and the food industry [3,4]. It is also an important cofactor for various hemoproteins, including hemoglobins, cytochrome P450 enzymes (P450s), peroxidases, and catalases, which catalyze a wide range of reactions [2,5]. As the chemical synthesis of heme and the extraction of heme from biological samples suffer from low-yielding, time-consuming, and eco-unfriendly properties [6,7], bio-synthesized heme is highly favored due to the advantages of simplicity, high efficiency, and eco-friendliness. Therefore, there is a great need for the efficient and stable synthesis of heme using microorganisms.

**Figure 1:**
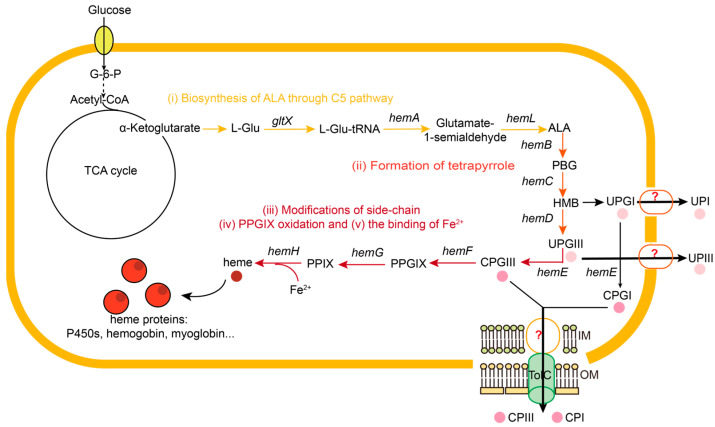
The biosynthesis pathway of heme. The biosynthesis of heme in *E. coli* is divided into the following parts: (i) biosynthesis of ALA through C5 pathway (yellow), (ii) formation of tetrapyrrole (orange), (iii) modification of side chain, (iv) PPGIX oxidation, and (v) the binding of Fe^2+^ (red). Solid lines indicate single reactions and dashed lines indicate more than two reactions. Black bold arrow indicates the efflux of porphyrin. L-Glu, L-glutamate; L-Glu-tRNA, L-glutamyl-tRNA; UPI/III, uroporphyrin I/III; CPI/III, coporphyrin I/III; OM, outer membrane; IM, inner membrane.

The biosynthesis of heme consists of the following steps: (i) the synthesis of the precursor 5-aminolevulinic acid (ALA), (ii) the formation of tetrapyrrole, (iii) the modification of the side chains, (iv) the oxidation of protoporphyrinogen IX (PPGIX), and (v) the binding of Fe^2+^ [1,4] (Figure 1). ALA is a crucial precursor for the synthesis of heme and is synthesized through the C4 or C5 pathway [8]. The C5 pathway, mainly found in archaea, plants, and most bacteria, converts L-glutamate (L-Glu) to ALA via glutamyl-tRNA synthetase (GluRS, encoded by *gltX*), glutamyl-tRNA reductase (GluTR, encoded by *hemA*), and glutamate-1-semialdehyde 2,1-aminomutase (GSAM, encoded by *hemL*). Notably, it has previously been reported that the C5 pathway is more efficient than the C4 pathway for the synthesis of ALA in *E. coli* [9]. ALA is then converted to the first tetrapyrrole intermediate, uroporphyrinogen III (UPGIII), through a series of reactions catalyzed by porphobilinogen synthase (PBGS, encoded by *hemB*), porphobilinogen deaminase (PBGD, encoded by *hemC*), and uroporphyrinogen III synthase (UROS, encoded by *hemD*). UPGIII is further modified and oxidated to protoporphyrin IX (PPIX) under the catalysis of uroporphyrinogen III decarboxylase (UROD, encoded by *hemE*), coproporphyrinogen III oxidase (CPO, encoded by *hemF*), and protoporphyrinogen oxidase (PPO, encoded by *hemG*). Finally, Fe^2+^ is incorporated into PPIX through ferrochelatase (FECH, encoded by *hemH*) to form heme [1,10,11].

There are a variety of microbial hosts used for the production of heme, including *E. coli*, *Saccharomyces cerevisiae*, *Corynebacterium glutamicum*, and *Rhodobacter sphaeroides* [9,12,13,14]. Among these, *E. coli* is the most widely used microorganism with the advantages of a clear genetic background, easy manipulation, and rapid growth [9,12,15,16,17,18,19,20,21]. The heme biosynthetic genes have generally been divided into several modules and overexpressed using plasmids with different copy numbers, respectively. By knocking out *pta*, *ildH*, and the putative heme degradation gene *yfeX*; overexpressing the heme exporter CcmABC; and optimizing the modular pathway, 239.2 mg/L of heme (of which 151.4 mg/L was secreted) was produced in fed-batch fermentation [9]. In addition, the expressional levels of the heme biosynthetic genes were further regulated by screening for suitable ribosome binding sites (RBSs) using a heme biosensor-guided screening strategy, resulting in 127.6 mg/L of heme in fed-batch fermentation [19]. The engineered strains with enhanced heme supply can also be further utilized for the synthesis of hemoproteins. The expression of heme biosynthetic genes (*hemAL*, *hemEFGH*) was used for the synthesis of one dye-decolorizing peroxidase (Dyp), six oxygen-transport proteins (hemoglobin, myoglobin, and leghemoglobin), and three P450 enzymes from CYP153A subfamily. The synthetic efficiencies of Dyp and oxygen transport proteins were increased by 42.3–107.0%, and the production of nonanedioic acid catalyzed by P450 enzymes was increased by 1.8- to 6.5-fold [18]. In another study, an engineered *E. coli* strain was constructed by the integrated expression of the necessary heme biosynthetic genes at proper ratios and the assembly of the rate-limiting enzymes using DNA-guided scaffolds. Combined with fine-tuned heme biosynthesis through heme biosensors and small regulatory RNA systems, the whole-cell catalytic activity of P450 enzyme BM3 was increased by 4.3-fold [17].

The efficient synthesis of heme is one of the major challenges for the application of heme and the production of active hemoproteins. Considering the complexity of heme biosynthesis in *E. coli*, which involves 10 genes, previous studies have highlighted the need to tune the expressional levels of heme biosynthetic genes to an optimal ratio for the efficient production of heme [9,18,19]. Notably, despite the expression of heme biosynthetic genes using plasmids with different copy numbers to regulate the expressional levels [9,15,16,17,19], previous studies have shown that maximizing the expression of all heme biosynthetic genes does not result in the highest titer of heme and may impose an additional metabolic burden [18]. Alternatively, enhancing the rate-limiting steps of biosynthetic pathway is considered an effective strategy and has been used to increase the production of hydroxytyrosol and β-alanine [22,23]. Therefore, modifying the rate-limiting steps in the heme biosynthetic pathway may be a more suitable approach to achieve efficient heme synthesis and reduce the metabolic burden. The assembly of cascade enzymes into multienzyme complexes is a common way to modify the rate-limiting steps [24,25]. Multienzyme complexes can form substrate channels to increase the efficiency of cascade reactions and avoid side reactions [26,27]. Scaffolds, including DNA scaffolds and protein scaffolds, can be used to assemble cascade enzymes [27,28,29]. It was previously reported that PBGS, PBGD, and UROS in heme synthesis were fused with the zinc-finger proteins ADB1, ADB2, and ADB3 and fixed to the corresponding sequences of DNA scaffolds. By adjusting the number and ratio of scaffolds, the titer of heme was increased to 1.4 mg/L [17]. Besides scaffolds, cascade enzymes can be assembled using scaffold-free methods including flexible linkers [30,31], hydrophobic proteins CipA or CipB [32,33], self-assembling amphipathic peptides (SAPs) [34], or self-assembled peptide pairs [35,36,37] to spontaneously form multienzyme complexes, avoiding the need to express additional scaffolds.

The engineering of transporter is also considered an effective strategy to increase the synthesis of heme [38]. It has previously been reported that excess porphyrin intermediates in *E. coli* are secreted by transporters such as TolC [39], which may reduce the efficiency of heme biosynthesis. Therefore, reducing the efflux of porphyrin intermediates may increase the metabolic flux of converted porphyrin intermediates for heme biosynthesis. In addition, multidrug efflux pumps have been shown to be involved in the efflux of porphyrins. In particular, a number of multidrug resistance-related transporters from the family of inner membrane ATP-binding cassette (ABC) transporters, which are often functionally redundant [40], have been shown to be involved in the efflux of porphyrins, such as MacAB in *E. coli* [41], PefCD in *Streptococcus agalactiae* [42], ABCB6 and ABCG2 in eukaryotes [43,44]. Furthermore, a transporter MsbA in *E. coli*, which belongs to the ABC transporter family and was predicted to be a multidrug efflux pump [45], shared 30% sequence similarity with the reported porphyrin efflux pump. Taken together, the mining and knockout of porphyrin transporters may facilitate the accumulation of porphyrin and the formation of heme.

The stable synthesis of heme is another major challenge in the application of heme for the production of active hemoproteins. Currently, plasmids with different copy numbers have been commonly used to express heme biosynthetic genes for the production of heme [9,16,18,21]. However, the instability of plasmids limits the stable synthesis of heme. In contrast, integrated expression of the key rate-limiting enzymes in the heme biosynthetic pathway seems to be a more favorable option due to its stability [46]. Although the integrated expression of the key rate-limiting enzymes in the heme biosynthetic pathway resulted in low levels of intracellular heme (0.3 mg/L), this strategy can increase the whole-cell catalytic activity of the P450 enzyme BM3 by 2.99-fold compared to plasmid-based expression of the key rate-limiting enzymes [17].

In this study, a plasmid-free heme-overproducing strain for the efficient and stable synthesis of heme was constructed to promote the production of active hemoproteins. First, the rate-limiting steps of heme biosynthesis were analyzed by examining the accumulation of porphyrin intermediates with the addition of ALA. In addition, various strategies, including promoter engineering, protein engineering, and scaffold-free assembly of the key rate-limiting enzymes were then employed to reduce the formation of the by-product UPGI. Next, the reported porphyrin transporter TolC was knocked out to increase the accumulation of intracellular porphyrin intermediates. Furthermore, to modify the heme biosynthetic genes in the genome of the *tolC* knockout strain, the rate-limiting enzymes HemC and HemD were assembled by RIAD-RIDD pairs to reduce the formation of by-product UPGI, and the promoters of the *hemE* and *hemH* genes encoding the rate-limiting enzymes were replaced by a strong constitutive promoter to increase the synthesis of intermediate CPGIII and heme. Moreover, the synthesis of heme was further enhanced by multicopy integrated expression of the *hemA* and *hemL* genes and knockout of the reported heme degradation gene *yfeX*. Finally, the engineered strain with enhanced and stable heme supply was used for the efficient synthesis of human hemoglobin and leghemoglobin.

## 2. Materials and Methods

### 2.1. Materials

First, 2 × TransStart^®^ FastPfu Fly PCR SuperMix (TransGen Biotech, Beijing, China) was used for PCR. A DNA purification kit and Plasmid Miniprep Purification kit were purchased from TransGen Biotech (Beijing, China). Hemin, 5-aminolevulinic acid hydrochloride (ALA), and coporphyrin (CP) were purchased from Sigma-Aldrich (St. Louis, MO, USA). Other chemicals were obtained from Sangon Biotech (Shanghai, China).

### 2.2. General Procedures

*E. coli* strain DH5α was used for DNA cloning. *E. coli* C41(DE3)-ΔhemA was used to test the rate-limiting steps in the heme biosynthetic pathway. *E. coli* strain C41(DE3) was used for the production of porphyrin and heme. All plasmids and strains used in this study are listed in Tables S1 and S2.

Luria broth (LB medium, 10 g/L NaCl, 10 g/L peptone, and 5 g/L yeast extract) was used for routine cloning, seed culture, and the production of porphyrin. LB medium supplemented with 20 mg/L of FeSO_4_∙7H_2_O (LB-Fe20) was used for the production of heme and hemoglobins.

Seed cultures were obtained by inoculating colonies of the recombinant strain on LB agar plates, and then 2.5 mL cultures of LB with appropriate antibiotics were cultivated in 12 mL test tubes in a rotary shaker for 12 h at 37 °C and 220 rpm. The seed cultures were then used for the production of porphyrin and heme. The inoculum used for the fermentation experiments was 2%. To maintain the growth of the C41(DE3)-ΔhemA strain in the seed cultures, 100 mg/L ALA was added. Antibiotics were added as supplements as follows: 50 μg/mL kanamycin; 100 μg/mL streptomycin; 34 μg/mL chloramphenicol; 100 μg/mL ampicillin.

### 2.3. The Construction of Plasmids and Strains

All constructed plasmids in this study were assembled using Gibson assembly, and the plasmids and primers used are listed in Tables S1 and S3.

To construct plasmid pRSFDuet-hemD (WT), the *hemD* gene was amplified from the genome of *E. coli* C41(DE3) using primers hemD-F/R and subcloned into *Nco* I/*BamH* I sites in plasmid pRSFDuet-1 to generate the plasmid pRSFDuet-hemD (WT). To construct the mutants of HemD, plasmid pRSFDuet-hemD (WT) was used as a template and amplified using primers 1-1-F/1-1-R to 9-2-F/9-2-R, to generate plasmids pRSFDuet-hemD-1-1 to 9-2, harboring the mutants of HemD.

To construct plasmids expressing *hemE*, the *hemE* gene was amplified from the genome of *E. coli* C41(DE3) using primers hemE (Ec)-F/R. The generated fragments were then subcloned into the *Nco* I/*BamH* I sites in plasmids pACYCDuet-1 and pRSFDuet-1 to generate the plasmids pACYCDuet-hemE (Ec) and pRSFDuet-hemE, respectively.

To construct plasmid pRSFDuet-hemC-hemD, in which the *hemC* and *hemD* genes were spaced by RBS (ATAAAAGGAGGAAAATAT), the *hemC* and *hemD* genes were amplified from the genome of *E. coli* C41(DE3) using primers hemC-F/R, hemD-F2/R, respectively. Overlap extension PCR was performed to fuse the fragments, and the resulting products were cloned into the *Nco* I/*BamH* I sites in plasmid pRSFDuet-1 to generate plasmid pRSFDuet-hemC-hemD.

To link HemC and HemD with flexible linkers L1 (GGGS), L2 (GGSGGGSG), and L3 (GGGGSGGGGSGGGGS), RBS between the *hemC* and *hemD* genes was replaced by flexible linkers L1, L2, and L3 with primers L1-F/R, L2-F/R, L3-F/R, respectively, which generated plasmids pRSFDuet-hemC-L1/2/3-hemD.

To assemble HemC and HemD with the hydrophobic proteins CipA and CipB, the *cipA cipB* genes were codon-optimized and synthesized by GenScript (Nanjing, China). CipA was directly fused into the N terminal of HemC and HemD, while CipB was fused into the N terminal of HemC and HemD with the ASASNGASA linker based on plasmid pRSFDuet-hemC-hemD. The *cipA* gene was amplified using primers CipA-C-F/R and CipA-D-F/R, respectively, while the *hemC* gene and the plasmid backbone were amplified using primers hemC-CipA-F/R and RSF-CipA-F/R, respectively, with plasmid pRSFDuet-hemC-hemD as a template. The resulting PCR products were assembled using Gibson assembly to generate plasmid pRSFDuet-cipA-hemC-hemD. Similarly, primers CipB-C-F/R, CipB-D-F/R, and hemC-CipB-F/R were used for the amplification of the *cipB* and *hemC* genes, respectively, while primers RSF-CipB-F/R were used for the amplification of the plasmid backbone. The resulting PCR products were assembled using Gibson assembly to generate plasmid pRSFDuet-cipB-hemC-hemD.

To assemble HemC and HemD with the self-assembling amphipathic peptide SAP(ANANARARANANARAR), SAP was fused into the N terminal of HemC and HemD, respectively, based on plasmid pRSFDuet-hemC-hemD. The *hemC* gene and the plasmid backbone were amplified using primers SAP-F/R and RSF-SAP-F/R with plasmid pRSFDuet-hemC-hemD as a template. The resulting PCR products were assembled using Gibson assembly to generate plasmid pRSFDuet-SAP-hemC-hemD.

To assemble HemC and HemD by self-assembled peptide pairs spytag/catcher, snooptag/catcher, and RIAD/RIDD, spycatcher and snoopcatcher were codon-optimized and synthesized by GenScript (Nanjing, China), while RIDD was amplified from a plasmid stored in our lab. The spytag/catcher and snooptag/catcher pairs were fused into the N or C terminals of HemC and HemD with the (GGGGS)_2_ linker, respectively, and generated 8 plasmids per pair. RIAD/RIDD were also fused into the N or C terminals of HemC and HemD with the (GGGGS)_3_ linker, respectively, and also generated 8 plasmids.

To construct plasmids pRSFDuet-Spy1-hemC-hemD to pRSFDuet-Spy8-hemC-hemD, firstly, spytag was fused into N or C terminals of HemC/D, respectively, using the primers YTC-F/R, CYT-F/R, YTD-F/R, and DYT-F/R using plasmid pRSFDuet-hemC-hemD as a template, which generated plasmids pRSFDuet-YTC, pRSFDuet-CYT, pRSFDuet-YTD, and pRSFDuet-DYT, respectively. To construct pRSFDuet-Spy1/2-hemC-hemD, then the spycatcher was amplified using primers YC-F/R using the synthesized gene as a template, and the plasmid backbones were amplified using primers RSF-YC-F/R with plasmids pRSFDuet-YTD and pRSFDuet-DYT as templates, respectively. The resulting PCR products were assembled using Gibson assembly to generate plasmids pRSFDuet-Spy1/2-hemC-hemD. Similarly, to construct pRSFDuet-Spy3/4-hemC-hemD, the spycatcher was amplified using primers CY-F/R with the synthesized gene as a template, and the plasmid backbones were amplified using primers RSF-CY-F/R with plasmids pRSFDuet-YTD and pRSFDuet-DYT as templates, respectively. To construct pRSFDuet-Spy5/6-hemC-hemD, the spycatcher was amplified using primers YD-F/R with the synthesized gene as a template, and the plasmid backbones were amplified using primers RSF-YD-F/R with plasmids pRSFDuet-YTC and pRSFDuet-CYT as templates, respectively. To construct pRSFDuet-Spy7/8-hemC-hemD, the spycatcher was amplified using primers DY-F/R with the synthesized gene as a template, and the plasmid backbones were amplified using primers RSF-DY-F/R with plasmids pRSFDuet-YTC and pRSFDuet-CYT as templates, respectively. The resulting PCR products were assembled using Gibson assembly. The plasmids pRSFDuet-Snoop1-hemC-hemD to pRSFDuet-Snoop8-hemC-hemD and pRSFDuet-RARD1-hemC-hemD to pRSFDuet-RARD8-hemC-hemD were generated the same way as pRSFDuet-Spy1-hemC-hemD to pRSFDuet-Spy8-hemC-hemD using the listed primers in Table S3.

For the modification of the *hemC* and *hemD* genes in the genome, to increase the expression of the *hemD* gene, a series of constitutive promoters (P_J23100_, P_J23104_, P_J23106_, P_J23114_, P_J23117_) and RBS (ATAAAAGGAGGAAAATAT) were added for the *hemD* gene in the genome, which generated GD1-5 strains using the modified CRISPR/Cas9 system [47]. To assemble HemC and HemD in the genome of the C41(DE3) strain, the assembled *hemC* and *hemD* fragments were amplified from plasmids pRSFDuet-hemC-L3-hemD, pRSFDuet-SPY1-hemC-hemD, and pRSFDuet-RARD6-hemC-hemD, respectively, to replace the previous *hemC* and *hemD* genes in the genome to generate GA1-3 strains; constitutive promoter P_J23104_ and RBS (ATAAAAGGAGGAAAATAT) were added for the *hemD* gene in the GA2/3 strains to generate the GA4/5 strains. All these strains were constructed using the modified CRISPR/Cas9 system [47].

To test the function of TolC for the efflux of porphyrin, the C41-ΔtolC strain was constructed by deleting the *tolC* gene in the C41(DE3) strain using the modified CRISPR/Cas9 system [47]. To test the function of other predicted ABC transporters, the C41-ΔybhRS, C41-ΔyddA, C41-ΔyojI, and C41-ΔmacAB strains were then constructed by deleting the *ybhRS*, *yddA, yojI*, *macAB* genes in the C41(DE3) strain using the modified CRISPR/Cas9 system [47], respectively. Meanwhile, the C41-ΔtolC-ΔybhRS, C41-ΔtolC-ΔyddA, C41-ΔtolC-ΔyojI, and C41-ΔtolC-ΔmacAB strains were constructed by deleting the *ybhRS*, *yddA, yojI*, *macAB* genes, respectively, in the C41-ΔtolC strain using the modified CRISPR/Cas9 system [47] to test the synergies of the transporters.

For the modification of rate-limiting steps in the genome of the C41-ΔtolC strain, the predicted promoter of *hemH* (80 bp upstream of the start codon) was replaced with constitutive promoters P_J23100_ and RBS (ATAAAAGGAGGAAAATAT) and generated the GH0 strain. To assemble HemC and HemD, the assembled *hemC* and *hemD* fragments were amplified from plasmids pRSFDuet-RARD6-hemC-hemD to replace the previous *hemC* and *hemD* genes in the genome of the GH0 strain, and constitutive promoter P_J23104_ and RBS (ATAAAAGGAGGAAAATAT) were added for the *hemD* gene to generate the GH1 strain. To enhance the expression of *hemE*, constitutive promoters P_J23100_ and RBS (ATAAAAGGAGGAAAATAT) were added for *hemE* in the genome of GH1 strain to generate the GH2 strain. The heme degradation *yfeX* gene was knocked out in the GH2 strain to generate the GH3 strain to enhance the concentration of heme. For the integrated expression of *hemAL* genes, the *hemA* and *hemL* genes were amplified using hemA-F/R and hemL-F/R, respectively, from the genome of the C41(DE3) strain, and constitutive promoters P_J23100_ and RBS (ATAAAAGGAGGAAAATAT) were added before the *hemA* gene. The amplified products were fused by overlap extension PCR to generate a P_J23100_-*hemA*-*hemL* fragment, and the constructed fragment was integrated to the *cheW*, *yciQ*, and *mbhA* locus. All these strains were constructed using the modified CRISPR/Cas9 system [47].

### 2.4. Culture Conditions and the Preparation for the Samples

For the strains without the integrated expression of *hemAL*, heme and its intermediate were detected by conducting flask cultivations in triplicate and with the addition of excessive ALA. First, 800 μL of the seed culture was transferred into 250 mL shaking flasks containing 40 mL LB or LB-Fe20 medium to facilitate the production of heme. Cultures were then incubated at 37 °C for 2 h, and 2 g/L ALA was added to the medium, followed by further incubation at 37 °C. For strains harboring plasmids, cultures were incubated at 37 °C until the OD_600_ reached 0.6–0.8, and 1 mM β-D-1-thiogalactopyranoside (IPTG) and 2 g/L ALA were added, followed by further incubation at 30 °C for 48 h. Antibiotics were added as supplements as follows: 50 μg/mL kanamycin; 34 μg/mL chloramphenicol. For strains with the integrated expression of *hemAL*, heme was detected by conducting flask cultivations in triplicate and without the addition of ALA. Next, 800 μL of the seed culture was transferred into 250 mL shaking flasks containing 40 mL LB-Fe20 medium and the cultures were then incubated at 37 °C for 48 h.

To detect ALA and PBG in the cultures by HPLC, 1 mL of cultures was harvested 48 h after incubation or every 12 h during fermentation for the rate-limiting step test, followed by cell disruption using Fastprep-24 (MP Biomedicals, Santa Ana, CA, USA). Cell debris was removed by centrifugation at 10,000× *g* for 10 min at 4 °C. Then, 5% trichloroacetic acid was added to the supernatant, and the tubes were incubated overnight at 4 °C and centrifuged at 10,000× *g* for 10 min at 4 °C. The supernatant was then used to examine the concentration of ALA and PBG by HPLC.

The intermediates uroporphyrinogen (UPG) and coporphyrinogen (CPG) were oxidized to uroporphyrin (UP) and coporphyrin (CP), respectively, during extraction [20]. Therefore, UP and CP were also detected in the cultures. To detect UP, CP, and PPIX in the cultures, 1 mL of cultures was harvested 48 h after incubation or every 12 h during fermentation for the rate-limiting step test, and then 20 μL of hydrochloric acid was added, followed by cell disruption using Fastprep-24 (MP Biomedicals, Santa Ana, CA, USA). Cell debris was removed by centrifugation at 10,000× *g* for 10 min at 4 °C. The supernatant was then used to examine the concentration of UP and CP by HPLC.

When testing the function of the predicted efflux pumps of porphyrin, to test the extracellular concentration of UP, CP, and PPIX, 1 mL of cultures was harvested and centrifuged at 8000× *g* for 10 min. The supernatant was then collected, and 20 μL of hydrochloric acid was added. The samples were then used to examine the concentration of UP, CP, and PPIX by HPLC. The cellular concentration of porphyrin is obtained by subtracting the concentration in the supernatant from the total amount. In addition, to assay porphyrin by LC-MS, 20 μL of hydrochloric acid was replaced by formic acid to a concentration of 6 M and followed by cell disruption using Fastprep-24 (MP Biomedicals, Santa Ana, CA, USA). Cell debris was removed by centrifugation at 10,000× *g* for 10 min at 4 °C. Acetonitrile was added into the supernatant to a concentration of 50% and incubated overnight at 4 °C, followed by centrifugation at 10,000× *g* for 10 min at 4 °C. The final supernatant was then used for LC-MS analysis.

For the detection of heme and ALA in the cultures by LC-MS, 1 mL of cultures was harvested 48 h after incubation and centrifuged at 10,000× *g* for 10 min. Cells were harvested and resuspended with 300 μL ammonia solution, followed by cell lysis with Fastprep-24 (MP Biomedicals, Santa Ana, CA, USA). Cell debris was removed by centrifugation at 10,000× *g* for 10 min at 4 °C. Next, 200 μL of the supernatant was added with 800 μL of acetonitrile, and then the tubes were incubated overnight at 4 °C, followed by centrifugation at 10,000× *g* for 10 min at 4°C. The final supernatant was then used for LC-MS analysis.

### 2.5. The Expression of Hemoglobins

To test the effect of the engineered strain on the synthesis of hemoglobins, the plasmid pETDuet-α-β (hHb) harboring the α and β subunits of human hemoglobin was transformed into original C41(DE3) and HEME2 strains, respectively, to generate the C41-hHb and HEME2-hHb strains. The plasmid pRSFDuet-sHb harboring leghemoglobin was transformed into the original C41(DE3) and HEME2 strains, respectively, to generate the C41-sHb and HEME2-sHb strains. The performance of hemoglobin synthesis was examined by conducting flask cultivations in triplicate. Next, 800 μL of the seed culture was transferred into 250 mL shaking flasks containing 40 mL LB-Fe20 medium with appropriate antibiotics. Cultures were incubated at 37 °C until the OD_600_ reached 0.6–0.8, and then 1 mM IPTG was added, followed by further incubation at 30 °C for 24 h.

To collect the pellets, 40 mL of cultures was harvested by centrifugation at 8000× *g* for 10 min and then resuspended in 20 mL PBS buffer (pH 7.4) and ultrasonicated on ice using an ultrasonic processor (Shanghai, China) under the following conditions: power, 38%; 2 s on /3 s off; 10 min. Cell debris was removed by centrifugation at 10,000× *g* for 10 min at 4 °C. Human hemoglobin and leghemoglobin were eluted with elution buffer (2 mL; PBS buffer, pH 7.4, 500 mM imidazole) using Ni-NTA His-Binding-resin (PointBio, Shanghai, China). The purified proteins were used for the determination of the concentration of proteins using the Bradford protein Assay Kit (Beyotime Biotech, Shanghai, China) and SDS-PAGE analysis. The concentration of hemoglobins was then calculated through grayscale analysis via SDS-PAGE. The peroxidase activities of purified proteins were detected using TMB Chromogen Solution (Beyotime Biotech, Shanghai, China). Enzyme activity (U/g DCW) was defined as 1 OD_370_ produced by per g dry cell weight of cultures. Dry cell weight was calculated according to the formula [48]. The heme content was then measured using a hemochrome binding assay [49,50].

### 2.6. Discovery Studio Analysis

The sequence of HemD was downloaded from UniProt (P09126). Hotspot Wizard 3.0 (http://loschmidt.chemi.muni.cz/hotspotwizard, accessed on 10 November 2023) [51] was used to analyze the amino acid frequency of HemD. The structure of HemD was downloaded from Alphafold2, and the HMB molecule was downloaded from PubChem. Molecular docking was performed using CDOCKER in Discovery Studio. The distance between Y158 of HemD and C20 of HMB was measured to select the best molecular docking result, which is shown in Table S4. Based on the result, the Calculate Mutation Energy (Binding) function was used to simulate the saturation mutagenesis of residues (not highly conservative) within a distance of 5 Å around the small molecule. The predicted mutants are shown in Table S5, and the mutation energy (<−0.5 kcal/mol) was the marker of increased affinity. The conservation of residues is shown in Table S6.

The structures of transporters MdlAB, YddA, YojHI, YbhFSR, YhhJ, and MacAB were downloaded from Alphafold2, and the CPGIII molecule was downloaded from PubChem. Molecular docking was performed using CDOCKER in Discovery Studio.

### 2.7. Analytical Methods

The optical density at 600 nm (OD_600_) was detected by a spectrophotometer (UVmini-1240, Shimadzu Corporation, Kyoto, Japan) to reflect cell growth. The concentrations of ALA, PBG, UP, CP, PPIX, and heme were analyzed using HPLC (Shimadzu, Kyoto, Japan) equipped with a ZORBAX Eclipse XDB-C18 (5 μm, 4.6 × 250 mm, Agilent, Santa Clara, CA, USA).

For the analysis of ALA and PBG, the method was based on that described by Park et al. through the derivatization of amino acids with OPA [52]. Mobile phase A contained 3.01 g sodium acetate in 1 L water with 200 μL triethylamine and 5 mL tetrahydrofuran (pH 7.2); mobile phase B contained 3.01 g sodium acetate in 200 mL water (pH 7.2), 400 mL methanol, and 400 mL acetonitrile. The parameters were set as follows: a flow rate of 0.8 mL/min; the mobile phase B ratio was 22% during the first 5 min, 30% at 29 min, 100% at 31.5 min, maintained at 100% to 35 min, 8% at 35.5 min, and maintained at 8% to 40 min; column temperature of 40 °C and monitored absorption wavelengths of 338 nm.

For the analysis of UP and CP, mobile phase A was 1M ammonium acetate aqueous solution (pH 5.15) with 8% acetonitrile; mobile phase B was methanol with 8% acetonitrile and 10% water. The parameters were set as follows: a flow rate of 0.8 mL/min; the mobile phase B ratio was 0 during the first 8 min, 65% at 38 min, maintained at 65% to 48 min, 0 at 49 min, and maintained at 0 to 55 min; column temperature of 40 °C and monitored absorption wavelengths of 404 nm.

For the analysis of heme and PPIX, mobile phase A was water with 0.1% trifluoroacetic acid; mobile phase B was methanol with 0.1% trifluoroacetic acid. The parameters were set as follows: a flow rate of 0.8 mL/min; a mobile phase B ratio of 30% for 1 min, 100% at 20 min, 100% at 35 min, and 30% at 37 min; column temperature of 40 °C and monitored absorption wavelengths of 400 nm.

For the analysis of ALA, heme, and PPIX by HPLC-MS, mobile phase A was 10 mM ammonium formate containing 0.1% formic acid and mobile phase B was acetonitrile containing 0.1% formic acid. First, 1 μL of samples were loaded onto an Agilent ZORBAX Eclipse Plus C18 column (2.1 × 50 mm, 1.8 μm), separated using Agilent 1290 Infinity II LC system, and quantified by an Agilent 6495C triple quadrupole mass spectrometer (Agilent, Santa Clara, CA, USA) equipped with an electrospray ionization (ESI) interface in the positive ion mode. The parameters were set as follows: a flow rate of 0.4 mL/min; mobile phase B ratio of 30% for 0.1 min, 40% at 0.4 min, 55% at 3 min, 100% at 4 min, 30% at 4.8 min, and maintained at 30% to 5.2 min. Mass transition, *m*/*z* 616.2 → 557.2 was selected to monitor heme, *m*/*z* 132.2 → 86.1 was selected to monitor ALA, and *m*/*z* 563.3 → 429.2 was selected to monitor PPIX. The capillary voltage was set at 2.5 kV. Nitrogen was used as the drying gas at a flow rate of 14 L/min at 200 °C. The nebulizer pressure was set at 24 psi.

## 3. Results and Discussion

### 3.1. Identification of the Rate-Limiting Steps in the Downstream Heme Biosynthetic Pathway

In *E. coli*, the synthesis of ALA is considered to be a key rate-limiting step in heme biosynthesis, and exogenous addition of ALA has been shown to be an effective strategy for increasing the synthesis of heme [8,53]. However, studies on the rate-limiting steps of the downstream heme biosynthetic pathway that converts ALA to heme are limited in *E. coli*. Metabolite accumulation is a commonly used method to identify rate-limiting steps [22,23,54,55]. To identify the rate-limiting steps of the downstream of heme that converts ALA to heme, a *hemA* knockout strain, C41(DE3)-ΔhemA, was employed [17], which relies on the exogenous addition of ALA to synthesize heme. The accumulation of intermediates suggests the rate-limiting steps in the downstream heme biosynthetic pathway, which is entirely dependent on the conversion of exogenous ALA addition.

**Figure 2:**
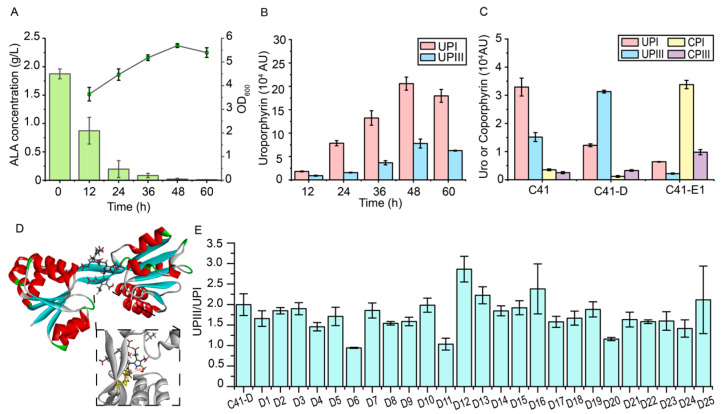
The identification of rate-limiting steps in the heme biosynthetic pathway and the modification of HemD (UROS). (**A**) The concentration of ALA and OD_600_ during the fermentation of the C41(DE3)-ΔhemA strain. (**B**) The titer of UPI and UPIII during the fermentation of C41(DE3)-ΔhemA strain with the addition of 2 g/L ALA. (**C**) The effect of overexpression of *hemD* and *hemE* on the accumulation of UP and CP. (**D**) The result of molecular docking for HemD and HMB. (**E**) The UPIII/UPI ratio of strains containing wild type HemD (C41-D strain) and different HemD mutants (D1–D25 strains). The peak area was normalized by OD_600_.

The accumulation of intermediates in the strain C41(DE3)-ΔhemA was monitored after the addition of 2 g/L ALA to the medium. After 48 h, 98.96% of the added ALA was consumed, but no accumulation of porphobilinogen (PBG) was detected (Figure 2A). Notably, hydroxymethylbilane (HMB) can be spontaneously converted to the by-product uroporphyrinogen I (UPGI) [56], and uroporphyrinogen (UPG) and coproporphyrinogen (CPG) were oxidized to uroporphyrin (UP) and coproporphyrin (CP), respectively, during extraction [20]. Therefore, uroporphyrin I (UPI) converted from UPGI and uroporphyrin III (UPIII) converted from UPGIII were monitored. The results showed that the titer of UPI in the strain C41(DE3)-ΔhemA was 1.0–4.0 times higher than that of UPIII during fermentation, suggesting that the lack of timely conversion of HMB to UPGIII resulted in the accumulation of the by-product UPGI (Figure 2B). Moreover, the results also revealed a significant accumulation of UPI and UPIII in the strain C41(DE3)-ΔhemA, but not of CPI and CPIII, suggesting that the conversion of UPG to CPG catalyzed by HemE is limited. Therefore, the conversion of HMB to UPGIII catalyzed by HemD and the conversion of UPGIII to CPGIII catalyzed by HemE may be the two key rate-limiting steps in the downstream heme biosynthetic pathway, and the limited conversion of HMB to UPGIII leads to the accumulation of by-product UPGI in the cell.

In addition, the binding of Fe^2+^ catalyzed by ferrochelatase (encoded by *hemH*) is considered to be a rate-limiting step in the heme biosynthetic pathway and may be regulated by Fe^2+^ and related transcription factors [19,57,58]. Therefore, the optimizations of these rate-limiting steps was performed, including the modification of *hemC* and *hemD* to reduce the formation of by-product UPGI and the enhancement of expressional levels of the *hemA*, *hemL*, *hemE*, and *hemH* genes.

### 3.2. The Engineering of UROS (HemD) to Reduce the Formation of By-Product UPGI

The above results showed that the limitation of the conversion of HMB to UPGIII catalyzed by uroporphyrinogen III synthase (UROS, HemD) leads to the accumulation of by-product UPGI in the cell. Previous studies have shown that uroporphyrinogen III decarboxylase (UROD, HemE) can catalyze the synthesis of CPGIII from UPGIII and can also catalyze the conversion of UPGI to another by-product, coproporphyrinogen I (CPGI) [21]. Therefore, to reduce the synthesis of these by-products, UPGI and CPGI, the effects of overexpression of the *hemD* and *hemE* genes on the accumulation of UPI and CPI in cells were first investigated. The plasmids pRSFDuet-hemD (WT) and pACYCDuet-hemE (Ec) were transformed into the C41(DE3) strain to generate strains C41-D and C41-E1, respectively. The synthesis of UPI and CPI in strains C41-D and C41-E1 were monitored after the addition of 2 g/L ALA to the medium (Figure 2C). The catalytic efficiency of HemD was assessed using the UPIII/UPI ratio, as it represents the ratio of HMB molecules catalyzed by HemD to those spontaneously converted. Compared to the C41(DE3) strain, the UPIII/UPI ratio was reversed in the C41-D strain, with a 4.55-fold increase. At the same time, the titer of CPI decreased by 66.6%, and the titer of CPIII increased by 30.3% compared to the C41(DE3) strain, indicating that the expression of the *hemD* gene can effectively reduce the production of the by-product UPGI and prevent its conversion to CPGI. In contrast, the titer of CPI and CPIII increased by 8.58-fold and 2.88-fold, respectively, in the C41-E1 strain compared to the C41(DE3) strain, suggesting insufficient selectivity for UPGI and UPGIII of endogenous HemE. The *hemE* genes from *Bacillus subtilis* and *C. glutamicum* were further introduced to increase the conversion of UPIII, but they exhibited lower efficiency for UPGI and UPGIII, although with better selectivity for UPIII, or they exhibited no effect (Figure S1). Therefore, the catalytic efficiency of HemD is crucial to reduce the synthesis of the by-products UPGI and CPGI.

To improve the catalytic efficiency of HemD, enzyme engineering of HemD was first attempted. UROS (HemD) generally consists of two domains, with the active site located in the cavity between the domains [59]. Due to the lack of a crystal structure of HemD in *E. coli*, the structure was downloaded from the Alphafold2 database. The conservation of HemD residues was then analyzed using Hotspot Wizard [51], and the binding pocket was defined using conserved residues near the cavity (R7, P8, S60, G88, R138, Y158). Subsequently, molecular docking of HMB and HemD was performed using CDOCKER in Discovery Studio. The distance between Y158 and C20 in HMB was used as a criterion to select the conformation for molecular docking, as the conserved tyrosine was involved in the formation of reaction intermediates [59]. Therefore, a conformation with a distance of 5.519 Å was selected (Figure 2D), and simulated saturation mutagenesis of 10 predicted non-conserved residues within 5 Å of the HMB was performed to calculate their effect on affinity, as an increase in affinity may facilitate the binding of the HMB to HemD and thus prevent its self-cyclization. A total of 25 mutants involving nine residues were screened, with mutations occurring predominantly on positively charged residues such as Arg, Lys, and His. It was hypothesized that the mutated residues could form additional electrostatic interactions with the carboxyl group of HMB.

To investigate the activities of HemD mutants, plasmids pRSF-hemD-1-1 to 9-2, containing the genes of 25 mutants, respectively, were transformed into the C41(DE3) strain to generate the strains D1-D25, respectively, while the C41-D strain was used as a control. The UPIII/UPI ratio was also used to reflect the catalytic efficiencies of HemD mutants (Figure 2E). Among the strains, the UPIII/UPI ratio showed an increase of 43.3%, 11.4%, and 19.3% in strains D12, D13, and D16, respectively (harboring L59V, Q61R and H62K mutants, respectively), compared to the C41-D strain. However, the titer of UPIII did not significantly increase (1.6% in the D13 strain, 12.5% in the D16 strain) or even decreased (13.8% in the D12 strain) compared to the C41-D strain, suggesting the lower activities of HemD mutants. Since then, considering the limited effect of the modification of HemD on the interaction with the HMB molecule for the synthesis of UPGIII, other approaches need to be applied to reduce the accumulation of UPI.

### 3.3. The Assembly of Rate-Limiting Enzymes HemC and HemD to Enhance the Synthesis of Intermediate UPGIII

Since the limited enhancement of UPIII by engineering of HemD, another approach of assembling the key enzymes into a multienzyme complex was tried. HemC and HemD were responsible for the synthesis of HMB and the conversion of HMB to UPGIII, respectively, and the assembly of HemC and HemD may form a substrate channel that facilitates the conversion of the generated HMB to UPGIII as in the previous study [17]. Considering the future modifications of *hemC* and *hemD* in the genome, different scaffold-free assembly methods were attempted to assemble HemC and HemD to enhance the synthesis of UPGIII.

**Figure 3:**
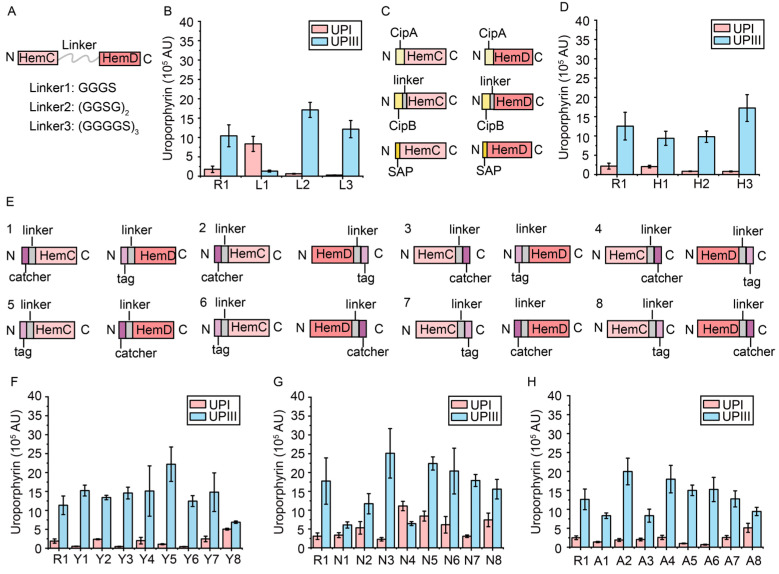
The assembly of HemC and HemD. (**A**) The fusion of HemC and HemD with different flexible linkers. (**B**) The titers of UPI and UPIII in L1-3 strains with the fusion of HemC and HemD using different flexible linkers. (**C**) The addition of hydrophobic protein CipA, CipB and self-assembling amphipathic peptides SAP to the N terminal of HemC and HemD. (**D**) The titers of UPI and UPIII in H1-3 strains with the addition of CipA, CipB, and SAP. (**E**) Different combinations of self-assembled peptide pairs at N or C terminals of HemC and HemD. (**F**–**H**) The titers of UPI and UPIII in the Y1-8, N1-8, and A1-8 strains with the addition of self-assembled peptide pairs spytag/catcher (**F**), snooptag/catcher, (**G**) and RIAD/RIDD (**H**). The peak area was normalized by OD_600_.

First, three flexible linkers were selected to fuse the key rate-limiting enzymes HemC and HemD (Figure 3A). Plasmids pRSFDuet-hemC-L1-hemD, pRSFDuet-hemC-L2-hemD, and pRSFDuet-hemC-L3-hemD, containing the different HemCD complex, were transformed into the C41(DE3) strain to generate strains L1–L3. As a control, plasmid pRSFDuet-hemC-hemD expressing *hemC* and *hemD* alone was transformed into the C41(DE3) strain to generate strain R1. The UPIII/UPI ratio was also used to reflect the catalytic efficiency of the HemCD complex in different recombinant strains. The results showed a 5.24-fold increase in the UPIII/UPI ratio and a 16.5% increase in the titer of UPIII in the L3 strain using the (GGGGS)_3_ linker compared to the R1 strain, indicating that the fused enzyme can reduce the spontaneous conversion of HMB (Figure 3B).

Subsequently, hydrophobic proteins and self-assembling amphipathic peptides (SAP, ANANARARANANARAR) were employed for the self-assembly of the key rate-limiting enzymes HemC and HemD (Figure 3C). To assemble HemC and HemD using hydrophobic proteins CipA, CipB, and SAP, plasmids pRSFDuet-CipA-hemC-hemD, pRSFDuet-CipB-hemC-hemD, and pRSFDuet-SAP-hemC-hemD, with CipA, CipB and SAP fused to the N-terminus of HemC and HemD, were transformed into the C41(DE3) strain to generate strains H1-H3. Compared to the R1 strain, the UPIII/UPI ratio in the H2 and H3 strains was increased by 0.96- and 2.58-fold, respectively, and the titer of UPIII was increased by 21.8% and 37.4%, respectively (Figure 3D), suggesting the synthesis of intermediate UPGIII was enhanced.

In addition, three self-assembled peptide pairs including spytag/catcher, snooptag/catcher, and RIAD/RIDD were also used to assemble the key rate-limiting enzymes HemC and HemD [35,36,37]. To assemble HemC and HemD, each pair of peptides was attached to the N-terminus or C-terminus of HemC and HemD, respectively, to generate eight assemblies (Figure 3E). To evaluate the effect of HemC and HemD assembly using self-assembled peptide pairs on the reduction of by-product UPGI synthesis, plasmids pRSFDuet-SPY1-hemC-hemD to pRSFDuet-SPY8-hemC-hemD, pRSFDuet-Snoop1-hemC-hemD to pRSFDuet-Snoop8-hemC-hemD, and pRSFDuet-RARD1-hemC-hemD to pRSFDuet-RARD8-hemC-hemD were transformed into the C41(DE3) strain to generate strains Y1-Y8, N1-N8, and A1-A8, respectively. The results showed that different combinations of peptide pairs had a significant effect on the catalytic efficiency (Figure 3F–H). The UPIII/UPI ratios in Y3 (HemC-spycatcher/spytag-HemD), A6 (HemC-RIAD/RIDD-HemD), and N3 (HemC-snoopcatcher/snooptag-HemD) strains were increased by 4.49, 3.40, and 0.93-fold, respectively, while the titer of UPIII was increased by 28.5%, 20.7% and 41.5%, respectively, compared to the R1 strain (Figure 3F–H).

In summary, in the assembly of HemC and HemD, the rate-limiting enzymes in the downstream heme biosynthetic pathway, based on the plasmid expression, can effectively reduce the formation of by-product UPGI and enhance the synthesis of intermediate UPGIII. Three methods of assembling HemC and HemD were obtained to significantly enhance the UPIII/UPI ratio and the synthesis of intermediate UPGIII, including the flexible linker L3 (HemC-(GGGGS)_3_-HemD), the spycatcher/spytag pair (HemC-spycatcher/spytag-HemD), and the RIAD-RIDD pair (HemC-RIAD/RIDD-HemD), and used for subsequent modifications of the *hemC* and *hemD* genes in the genome.

### 3.4. Regulation of the Expressions of hemC and hemD Genes in the Genome to Enhance the Synthesis of Intermediate UPGIII

**Figure 4:**
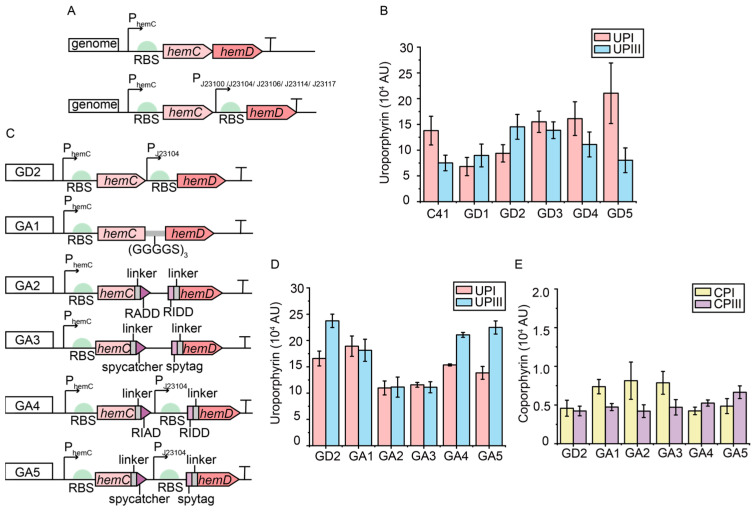
The modification of *hemC* and *hemD* genes in the genome of C41(DE3) strain. (**A**) The addition of different constitutive promoters (P_J23100_, P_J23104_, P_J23106_, P_J23114_ and P_J23117_) for the *hemD* gene. (**B**) The titers of UPI and UPIII in GD1-5 strains with the addition of different constitutive promoters for *hemD*. (**C**) The assembly of HemC and HemD in the genome through flexible linker, spytag/catcher, and RIAD/RIDD pairs. (**D**,**E**) The titers of UPI and UPIII (**D**) and of CPI and CPIII (**E**) in GA1-5 strains after the assembly of HemC and HemD. The peak area was normalized by OD_600_.

To regulate the expressions of the *hemC* and *hemD* genes in the genome of the C41(DE3) strain to enhance the synthesis of intermediate UPGIII, the location of the *hemC* and *hemD* genes in the genome of the C41(DE3) strain was first analyzed. It was found that the *hemC* and *hemD* genes were expressed under the same promoter, and the *hemD* gene was located in the downstream site of the *hemC* gene. In general, the expression of downstream genes will be lower in polycistronic mRNA [60]. The expressional level of the *hemD* gene was found to have a major effect on the synthesis of by-product UPGI. Therefore, to increase the expressional level of the *hemD* gene on the chromosome, a series of constitutive promoters with different strengths (P_J23100_, P_J23104_, P_J23106_, P_J23114_, and P_J23117_) were introduced to facilitate the transcription of the *hemD* gene, resulting in strains GD1-5 (Figure 4A). Except for the strain GD5 (*hemC*-P_J23117_-*hemD*), the UPIII/UPI ratio in the other recombinant strains increased by 26.4–141.2% compared to the control strain C41(DE3) (Figure 4B). Notably, the UPIII/UPI ratio reached 1.55 and the titer of UPIII was increased by 93.2% in strain GD2, expressing the *hemD* gene under the control of promoter P_J23104_ with medium strength, suggesting that increasing the expressional level of the HemD gene is an effective strategy to improve the synthesis of UPIII.

Subsequently, the assembly of HemC and HemD in the genome was investigated to enhance the synthesis of intermediate UPIII using the selected methods, including the flexible linker L3 (HemC-(GGGGS)_3_-HemD), the spycatcher/spytag pair (HemC-spycatcher/spytag-HemD), and the RIAD-RIDD pair (HemC-RIAD/RIDD-HemD), generating strains GA1-GA5 (Figure 4C). Compared to the GD2 strain, the titer of UPI was reduced by 16.5% in the GA5 strain (*hemC*-RIAD-P_J23104_-RIDD-*hemD*), suggesting that the assembly of HemC and HemD can further reduce the synthesis of by-product UPGI (Figure 4D). Despite a slight decrease of 5.3% in the titer of UPIII, the titer of CPIII increased by 57.2% in the GA5 strain compared to the GD2 strain, suggesting that the assembly of HemC and HemD is beneficial for the synthesis of CPGIII, which is converted from UPGIII by HemE (Figure 4E). Overall, the use of constitutive promoter P_J23104_ to enhance the expressional level of the *hemD* gene at the genomic level and the use of self-assembling peptide RIAD-RIDD for the assembly of HemC and HemD can effectively enhance the synthesis of intermediate UPGIII and reduce the formation of by-product UPGI.

### 3.5. Reduce the Efflux of Porphyrin Intermediates by Transporter Engineering

The previous studies have shown that ALA supplementation leads to the accumulation of many porphyrin intermediates—UPGIII, CPGIII, and PPIX—in *E. coli*. Thus, the excess porphyrin intermediates are secreted by transporters [39,41]. To further increase the metabolic flux of porphyrin intermediates for the synthesis of heme, the intracellular supply of porphyrin compounds was increased by reducing the efflux of porphyrin intermediates.

**Figure 5:**
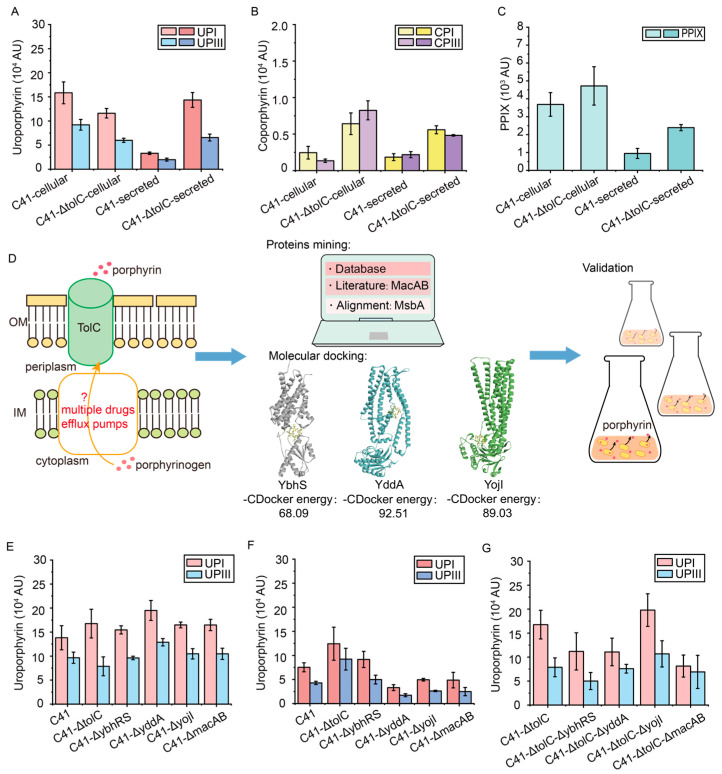
The knockout of porphyrin transporter TolC and the mining of other efflux pumps of porphyrin. (**A**–**C**) The intracellular and extracellular titers of UPI and UPIII (**A**), CPI and CPIII (**B**), and PPIX (**C**) after the knockout of the *tolC* gene. (**D**) The scheme of mining of other efflux pumps of porphyrin in the inner membrane. The multiple drug efflux pumps in the ABC transporter family were searched. Besides the previously reported MacAB, the three proteins YbhS, YddA, and YojI were successfully docked with CPGIII. The transporters were knocked out, and the efflux of porphyrin was then validated by shaking-flask culture. (**E**,**F**) The intracellular (**E**) and extracellular (**F**) titers of UPI and UPIII in transporter-knockout strains. (**G**) The intracellular titers of UPI and UPIII in strains with the deletion of both the *tolC* gene and the predicted ABC transporter. The peak area was normalized by OD_600_.

The outer membrane protein TolC has been reported to be involved in the efflux of various porphyrin intermediates in *E. coli*, and the knockout of the *tolC* gene resulted in the accumulation of CPGIII [39]. To verify the effect of *tolC* knockout on the intracellular titer of porphyrin intermediates, the strain C41-ΔtolC was generated. The results showed that the intracellular titers of UPI and UPIII were reduced by 26.7% and 34.9%, respectively, compared to the C41(DE3) strain (Figure 5A). In addition, the intracellular titers of CPI and CPIII in the C41-ΔtolC strain increased by 1.62-fold and 5.11-fold, respectively, compared to the C41(DE3) strain (Figure 5B), which was consistent with a previous study [39]. Furthermore, the intracellular titers of PPIX also increased by 28.2% compared to the C41(DE3) strain (Figure 5C). Unexpectedly, despite the effective cellular accumulation of CP, the extracellular titers of both CP and UP increased (Figure 5A,B). It suggests that there is other mechanism for the efflux of porphyrins, and more efflux pumps need to be further explored. Since TolC is located in the outer membrane, some porphyrins can still be secreted into the periplasm through transporters in the inner membrane [39].

The combination of transporter mining and molecular docking is an effective method for transporter identification [38] (Figure 5D). Among various efflux pumps in the inner membrane, ABC transporters often function as multidrug efflux pumps in *E. coli* [40,41,61] and were predicted to function as the potential porphyrin transporters. Most porphyrin transporters belong to this family, including the PPIX transporter MacAB and the multidrug efflux pump MsbA with 30% sequence similarity to the known transporters [41,42,43,44]. Five ABC transporters predicted to be multidrug efflux pumps in *E. coli* were then selected from the TransportDB2 database (http://www.membranetransport.org/, accessed on 20 April 2024) [62] and previous studies [39,40], including mdlAB, YddA, YojHI, YbhFSR, and YhhJ. Molecular docking of the selected transporters with CPGIII was then performed using Discovery Studio. In addition to MacAB and MsbA, YddA, YbhS, and YojI were successfully docked with CPGIII and predicted to be potential porphyrin transporters. Among them, MsbA is involved in lipid transport and the formation of lipopolysaccharide, which is essential for the viability of *E. coli* [45,63]. Therefore, the other four transporters, MacAB, YddA, YbhS, and YojI were knocked out to investigate their involvement in porphyrin transport, and strains C41-ΔybhRS, C41-ΔyddA, C41-ΔyojI, and C41-ΔmacAB were generated. Among these engineered strains, the extracellular titers of UPI and UPIII were significantly reduced by 56.1% and 59.5%, respectively, in the C41-ΔyddA strain compared to the C41(DE3) strain, accompanied by a 41.0% and 33.4% increase in their intracellular titers (Figure 5E,F). It suggests that YddA may be involved in the efflux of UP. All the knockouts of these transporters had no significant effect on the accumulation of CP (Figure S2).

The synergistic effect of knocking out the *tolC* gene and the genes encoding other transporters was further verified by constructing strains C41-ΔtolC-ΔybhRS, C41-ΔtolC-ΔyddA, C41-ΔtolC-ΔyojI, and C41-ΔtolC-ΔmacAB. However, there was no further increase in the intracellular titer of UP in the C41-ΔtolC-ΔyddA strain compared to the control strain C41-ΔtolC, whereas the intracellular titers of UPI and UPIII in the C41-ΔtolC-ΔyddA strain were reduced by 34.0% and 3.8%, respectively (Figure 5G). These results were consistent with the previous results that endomembrane transporters may be highly redundant and more endomembrane transporters need to be identified [39]. Therefore, considering the efficient increase in CPIII titer, the strain C41-ΔtolC was selected as the starting strain for subsequent metabolic engineering to modify the heme biosynthetic pathway.

### 3.6. Construction of a Plasmid-Free Escherichia coli Strain with Enhanced Heme Supply

**Figure 6:**
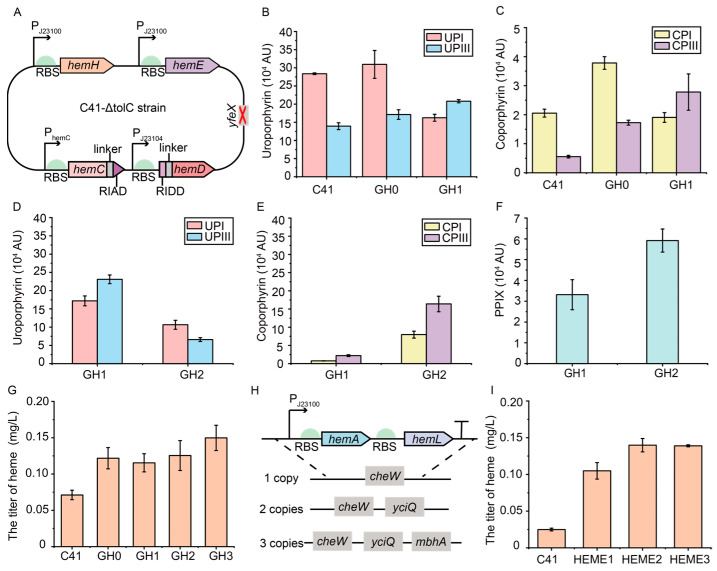
Modifications of rate-limiting steps in C41-ΔtolC strain. (**A**) Modifications of heme biosynthetic genes in genome of C41-ΔtolC strain. (**B**,**C**) Titers of UPI and UPIII (**B**) and CPI and CPIII (**C**) in C41(DE3), GH0, and GH1 strains. (**D**–**F**) Titers of UPI and UPIII (**D**), CPI and CPIII (**E**), and PPIX (**F**) in GH1 and GH2 strains. (**G**) Titers of heme in C41(DE3) and GH0-3 strains with addition of 2 g/L ALA. (**H**) Integration of fragment P_J23100_-*hemA-hemL* into *cheW*, *yciQ*, *mbhA* loci to generate strains with different copy numbers of *hemAL* gene. (**I**) Titers of heme in C41(DE3) and HEME1-3 strains. Peak area was normalized by OD_600_.

To achieve the stable synthesis of heme, the heme biosynthetic genes in the C41-ΔtolC strain were optimized at the genomic level (Figure 6A). Firstly, the reported crucial rate-limiting step in heme biosynthesis, the binding of Fe^2+^ catalyzed by ferrochelatase (HemH), was modified. The efficiency of HemH directly affects the production of heme, and the expressional level of the *hemH* gene was subject to complex transcriptional regulation [19,57,58]. To increase the expressional level of the *hemH* gene and to avoid potential regulation, the original promoter of the *hemH* gene was replaced with a strong constitutive promoter P_J23100_, and strain GH0 was generated. Subsequently, to reduce the synthesis of by-product UPGI, the *hemD* gene was expressed using the medium-strength constitutive promoter P_J23104_, and then the self-assembling peptide RIAD-RIDD was used to assemble HemC and HemD to obtain the strain GH1. The synthesis of heme was carried out using LB-Fe20 medium. After the addition of 2 g/L ALA to the medium, the titer of heme in both GH0 and GH1 strains reached 0.12 mg/L compared to the control strain C41(DE3) (0.07 mg/L) (Figure 6G). It suggests that the enhanced expressional level of the *hemH* gene effectively increased the synthesis of heme. Compared to the GH0 strain, after assembling HemC and HemD with the RIAD-RIDD pairs, the titer of by-product UPI exhibited a 47.5% decrease in the GH1 strain, while the titers of UPIII and CPIII were increased by 21.4% and 61.1%, respectively. It means that the conversion from HMB to UPGIII and UPGIII to CPGIII was enhanced (Figure 6B,C).

The titer of CP in the strain GH1 was lower than that of UP, which was consistent with the previous results in the control strain C41(DE3) (Figure 6B,C). Previous studies have shown that the overexpression of the *hemE* gene has a positive effect on heme accumulation [18], while the individual overexpression of the *hemF* and *hemG* genes has a relatively slight effect, and the overexpression of the *hemF* gene affects the activity of HemB [9]. To facilitate the conversion of UPGIII to CPGIII, the promoter of *hemE* in the GH1 strain was replaced with the strong constitutive promoter P_J23100_ to increase its expression, generating in the strain GH2. Compared with the GH1 strain, the GH2 strain showed a 71.4% decrease in the titer of UPGIII and a 6.50-fold increase in the titer of CPIII. It indicates that UPGIII could be efficiently converted to CPGIII (Figure 6D,E). In addition, the titer of PPIX was increased by 78.6% in the GH2 strain compared to the GH1 strain. It suggests that the further conversion of CPGIII to PPIX was also enhanced with a heme titer of 0.13 mg/L (Figure 6F,G). The reported *yfeX* gene related to heme degradation [9] was further knocked out in the GH2 strain to generate the GH3 strain. A heme titer of 0.15 mg/L was achieved in the GH3 strain, an increase of 110.5% and 19.4% compared to the C41(DE3) and GH2 strains, respectively (Figure 6G).

To achieve the efficient de novo synthesis of heme, the synthetic pathway of heme precursor ALA was enhanced. The P_J23100_-*hemA*-*hemL* fragment was integrated into the previously used *cheW* locus [17] and the reported loci *yciQ* and *mbhA* [64] in the GH3 strain, respectively, to generate strains HEME1-3, which contained 1, 2, and 3 copies of *hemAL* genes, respectively (Figure 6H). The synthesis of heme was carried out using LB-Fe20 medium. After 24 h of fermentation, the titer of heme reached 0.14 mg/L in the HEME2 strain containing two copies of *hemAL* genes, which was 4.60-fold higher than that of the C41(DE3) strain (0.02 mg/L) and 33.3% higher than that of the HEME1 strain (0.10 mg/L). In addition, the titer of heme in the HEME3 strain was similar to the titer in the HEME2 strain (0.14 mg/L) (Figure 6I). Therefore, the HEME2 strain, a plasmid-free engineered *E. coli* strain with enhanced heme supply, was constructed at the genomic level. Overall, although plasmid-based expression has been widely used in the synthesis of heme [9,14,15,16,17,18,19,20,21], plasmid-free systems are genetically more stable than plasmid-based expression, and the supply of heme can be enhanced by using plasmid-free systems without the addition of antibiotics [46]. Compared with a previous study that used the T7-lac promoter to express heme biosynthetic genes to construct a plasmid-free *E. coli* [17], constitutive promoters were used to construct the plasmid-free *E. coli* HEME2 strain in this study, thus avoiding the need to add the costly inducer IPTG for the stable synthesis of heme.

### 3.7. Efficient Synthesis of High-Active Hemoglobins Using the Strain HEME2 with Stable and Enhanced Heme Supply

**Figure 7:**
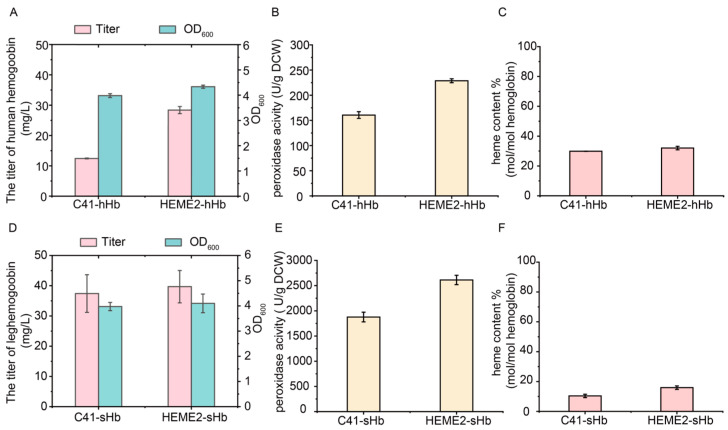
The expression of hemoglobins using the strain HEME2. (**A**) The titers (mg/L) of human hemoglobin and the values of OD_600_ in different strains. (**B**) The peroxidase activities (U/g DCW) of human hemoglobin in different strains. (**C**) The heme contents (mol/mol hemoglobin) of human hemoglobin in different strains. (**D**) The titers (mg/L) of leghemoglobin and the values of OD_600_ in different strains. (**E**) The peroxidase activities (U/g DCW) of leghemoglobin in different strains. (**F**) The heme contents (mol/mol hemoglobin) of leghemoglobin in different strains.

The constructed strain HEME2 and the original strain C41(DE3) were evaluated for the efficient synthesis of active hemoglobins. The gene encoding human hemoglobin, a tetramer composed of α and β subunits [65], was expressed using the plasmid pETDuet-1. The plasmid pETDuet-hHb was transferred into the strains C41(DE3) and HEME2, respectively, to generate the strains C41-hHb and HEME2-hHb. The cells were harvested and the human hemoglobin was purified after 24 h of induced expression. The titer of human hemoglobin reached 28.4 mg/L in the HEME2-hHb strain, which was 1.29-fold higher than that in the C41-hHb strain (Figure 7A and Figure S3A). In addition, as it was previously reported that active hemoglobin could function as a peroxidase [66], the enzymatic activity of human hemoglobin was detected. The result showed that the peroxidase activity of human hemoglobin reached 228.7 U/g DCW in the HEME2-hHb strain, which was 42.4% higher than that in the C41-hHb strain (Figure 7B). Furthermore, the heme content of human hemoglobin, reflecting the proportion of heme per mol of heme proteins [49], was similar in the C41-hHb and HEME2-hHb strains at 29.9% and 32.0%, respectively (Figure 7C). It suggests that the increase in peroxidase activity was driven by the increase in the synthesis and stability of human hemoglobin.

The expression of leghemoglobin was then tested using the plasmid pRSFDuet-1 in the C41(DE3) and HEME2 strains, generating the recombinant strains C41-sHb and HEME2-sHb. The cells were harvested and the leghemoglobin was purified after 24 h of induced expression. Although the titers of leghemoglobin in the C41-sHb and HEME2-sHb strains were similar at 37.4 mg/L and 39.7 mg/L, respectively (Figure 7D and Figure S3B), the peroxidase activity of leghemoglobin in the HEME2-sHb strain reached 2613.0 U/g DCW, which was 39.2% higher than that in the C41-sHb strain (Figure 7E). This result could be attributed to the increase in heme content of leghemoglobin to 15.9% in the HEME2-sHb strain, which was 53.4% higher than that of leghemoglobin in the C41-sHb strain (Figure 7F). Therefore, the constructed HEME2 strain can effectively enhance the synthesis of active hemoglobins.

Consistent with previous findings that the whole-cell catalytic activities of P450s were enhanced by increasing intracellular heme supply with the integrated expression of heme biosynthetic genes [17], the titer of human hemoglobin and the heme content of leghemoglobin were effectively increased in the constructed plasmid-free HEME2 strain compared to the original strain C41(DE3) in our study, suggesting the potential for efficient synthesis of high-active hemoglobins using the strain HEME2 with stable and enhanced heme supply.

## 4. Conclusions

In this study, a plasmid-free engineered *E. coli* strain with enhanced heme supply was constructed by modifying the rate-limiting steps in heme biosynthesis. Firstly, based on previous studies and the accumulation of heme intermediates with the addition of ALA in the C41-ΔhemA strain, the rate-limiting steps in heme biosynthesis were analyzed: (i) the synthesis of ALA, (ii) the conversion of HMB to UPGIII, (iii) the conversion of UPGIII to CPGIII, and (iv) the binding of Fe^2+^ to PPIX. Among these steps, the untimely conversion of HMB to UPGIII was the main reason for the formation of the by-product UPGI. Subsequently, to modify the heme biosynthetic genes in the genome, the self-assembled peptide pair RIAD-RIDD was used to assemble HemC and HemD, which increased the synthesis of intermediate UPGIII. In addition, the knockout of porphyrin transporter TolC increased the accumulation of the intracellular porphyrin intermediate CPGIII. Moreover, the strong constitutive promoter P_J23100_ was used to replace the promoters of the *hemE* and *hemH* genes in the genome, respectively, to increase the synthesis of intermediate CPGIII and heme. The *yfeX* gene related to heme degradation was knocked out to further increase the accumulation of heme. Furthermore, the *hemA* and *hemL* genes were integrated into the *cheW* and *yciQ* locus to promote ALA synthesis. Finally, the HEME2 strain was obtained with a heme titer of 0.14 mg/L, which was 4.60-fold higher than that in the C41 (DE3) strain (0.02 mg/L). The HEME2 strain was used for the synthesis of human hemoglobin and leghemoglobin. The titer of human hemoglobin and the peroxidase activity were 1.29-fold (28.4 mg/L) and 42.4% (228.7 U/g DCW) higher, respectively, in the HEME2-hHb strain than in the control strain C41-hHb. The peroxidase activity and heme content of leghemoglobin were increased by 39.2% (2613.0 U/g DCW) and 53.4% (15.9%), respectively, in the HEME2-sHb strain compared to the control strain C41-sHb. In conclusion, the constructed plasmid-free HEME2 strain was able to stably and efficiently provide heme to promote the synthesis of active hemoproteins.

## Data Availability

The datasets are available from the corresponding author on reasonable request.

## Supplementary Materials

The following supporting information can be downloaded at: https://www.mdpi.com/article/10.3390/metabo15030151/s1, Figure S1: Effect of overexpression of *hemE* from different bacterial sources on the accumulation of UP and CP. *hemE* genes from *Escherichia coli*, *Bacillus subtilis*, and *Corynebacterium glutamicum* were expressed in strains C41-E1, C41-E2 and C41-E3, respectively; Figure S2: The intracellular (A) and extracellular (B) titers of CPI and CPIII in transporter-knockout strains.; Figure S3: The SDS-PAGE results of purified human hemoglobin. (A) and leghemoglobin (B) obtained in different engineered strains (M: Marker; Lines 1-2: C41-hHb, HEME2-hHb, Lines 3–4: C41-sHb, HEME2-sHb); Table S1: Plasmids used in this study; Table S2: Strains used in this study; Table S3: Primers used in this study; Table S4: The result of molecular docking for HemD and HMB; Table S5: The mutations of HemD predicted by simulate saturation mutagenesis of residues within a distance of 5 Å around the small molecule; Table S6. The conservation of HemD residues within a distance of 5 Å around HMB molecule analyzed by Hotspot Wizard (The mutable score reflects the conservation of residues, with a lower score indicating higher conservation. Mutable score under 3 was considered highly conserved); Supplementary notes: Gene sequences used in this study.
