# Supplementary material for: Construction of a Plasmid-Free Escherichia coli Strain with Enhanced Heme Supply to Produce Active Hemoglobins
Source: Metabolites. 2025 Feb 23;15(3):151. doi: 10.3390/metabo15030151 (PMC11943725; doi:10.3390/metabo15030151)
Supplement: Supplementary file 1 [file metabolites-15-00151-s001.zip › metabolites-3485879-supplementary.pdf]

Supplementary Materials

# Construction of a Plasmid-Free *Escherichia coli* Strain with Enhanced Heme Supply to Produce Active Hemoglobins

**Zihan Zhang [1,2,†], Baodong Hu [1,2,†], Jingwen Zhou [1,2], Jianghua Li [1,2], Jian Chen [1,2], Guocheng Du [1,2,3,*] and Xinrui Zhao [1,2,*]**

[1] Science Center for Future Foods, Jiangnan University, 1800 Lihu Road, Wuxi 214122, China
[2] Key Laboratory of Industrial Biotechnology, Ministry of Education, School of Biotechnology, Jiangnan University, 1800 Lihu Road, Wuxi 214122, China
[3] Key Laboratory of Carbohydrate Chemistry and Biotechnology, Ministry of Education, Jiangnan University, 1800 Lihu Road, Wuxi 214122, China
* Correspondence: gcdu@jiangnan.edu.cn (G.D.), zhaoxinrui@jiangnan.edu.cn (X.Z.)
† These authors contributed equally to this work.

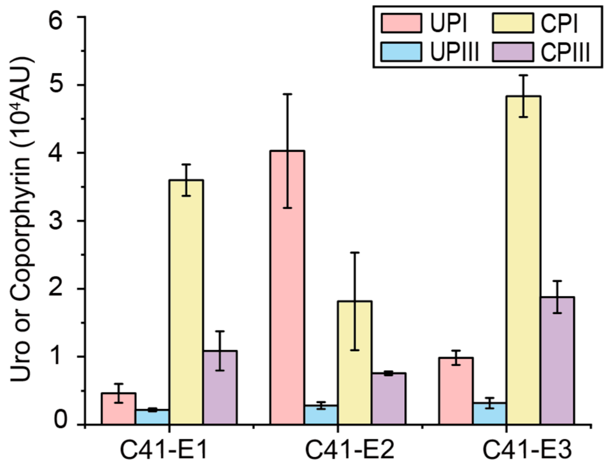


**Figure S1.** Effect of overexpression of *hemE* from different bacterial sources on the accumulation of UP and CP. *hemE* genes from *Escherichia coli*, *Bacillus subtilis*, and *Corynebacterium glutamicum* were expressed in strains C41-E1, C41-E2 and C41-E3, respectively.

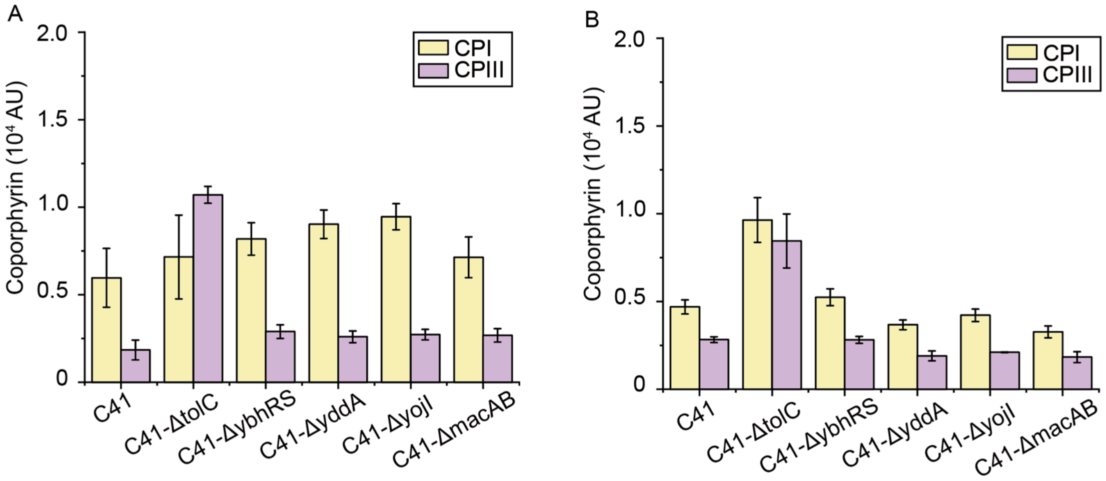


**Figure S2.** The intracellular (A) and extracellular (B) titers of CPI and CPIII in transporter-knockout strains.

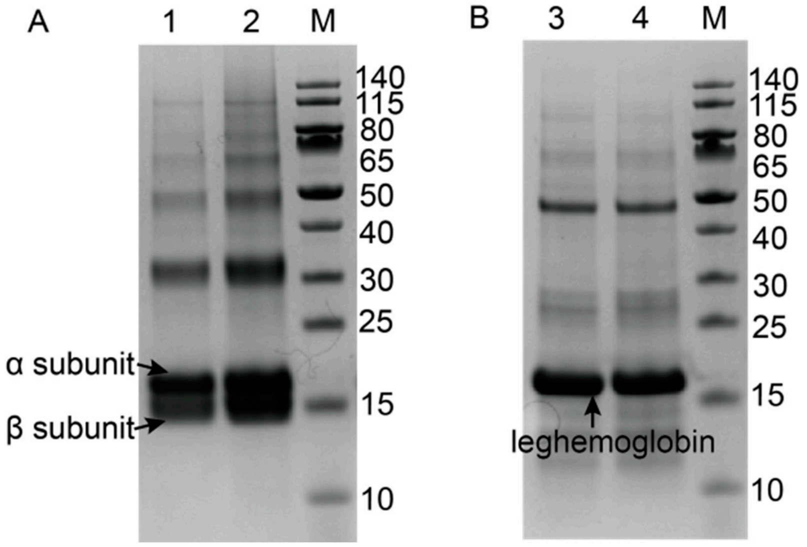


**Figure S3.** The SDS-PAGE results of purified human hemoglobin (A) and leghemoglobin (B) obtained in different engineered strains (M: Marker; Lines 1-2: C41 -hHb, HEME2-hHb, Lines 3-4: C41-sHb, HEME2-sHb)

**Table S1.** Plasmids used in this study

| Plasmids | Descriptions | Source |
|---|---|---|
| pCDFDuet | Expression vector, CloDF13 *ori*, double T7 *lac* promoters; $Sm^R$ | Novagen |
| pACYCDuet-1 | Expression vector, p15A *ori*, double T7 *lac* promoters; $Cm^R$ | Novagen |
| pRSFDuet-1 | Expression vector, ColE1 *ori*, double T7 *lac* promoters; $Km^R$ | Novagen |
| pEcCas9 | *repA101*(Ts) *kan Pcas-cas9* $P_{araB}$*-Red* $lacI^q$ PrhaB-sgRNA-*pMB1*, *sacB*; $Km^R$ | lab storage |
| ptargetF | pMB1 *aadA* sgRNA-*pMB1*; $Sm^R$ | lab storage |
| pRSFDuet-hemD(WT) | pRSFDuet-1 derivative, *E. coli* C41(DE3) *hemD*; $Km^R$ | this study |
| pRSFDuet-hemE | pRSFDuet-1 derivative, *E. coli* C41(DE3) *hemE*; $Km^R$ | this study |
| pACYCDuet-hemE(Ec) | pACYCDuet-1 derivative, *E. coli* C41(DE3) *hemE*; $Cm^R$ | this study |
| pACYCDuet-hemE(Bs) | pACYCDuet-1 derivative, *Bacillus subtilis hemE*; $Cm^R$ | this study |
| pACYCDuet-hemE(Cg) | pACYCDuet-1 derivative, *Corynebacterium glutamicum hemE*; $Cm^R$ | this study |
| pRSFDuet-hemD-1-1 | pRSFDuet-1 derivative, *E. coli* C41(DE3) *hemD* mutation 1-1; $Km^R$ | this study |
| pRSFDuet-hemD-1-2 | pRSFDuet-1 derivative, *E. coli* C41(DE3) *hemD* mutation 1-2; $Km^R$ | this study |
| pRSFDuet-hemD-1-3 | pRSFDuet-1 derivative, *E. coli* C41(DE3) *hemD* mutation 1-3; $Km^R$ | this study |
| pRSFDuet-hemD-2-1 | pRSFDuet-1 derivative, *E. coli* C41(DE3) *hemD* mutation 2-1; $Km^R$ | this study |
| pRSFDuet-hemD-2-2 | pRSFDuet-1 derivative, *E. coli* C41(DE3) *hemD* mutation 2-2; $Km^R$ | this study |
| pRSFDuet-hemD-3-1 | pRSFDuet-1 derivative, *E. coli* C41(DE3) *hemD* mutation 3-1; $Km^R$ | this study |
| pRSFDuet-hemD-3-2 | pRSFDuet-1 derivative, *E. coli* C41(DE3) *hemD* mutation 3-2; $Km^R$ | this study |
| pRSFDuet-hemD-3-3 | pRSFDuet-1 derivative, *E. coli* C41(DE3) *hemD* mutation 3-3; $Km^R$ | this study |
| pRSFDuet-hemD-3-4 | pRSFDuet-1 derivative, *E. coli* C41(DE3) *hemD* mutation 3-4; $Km^R$ | this study |
| pRSFDuet-hemD-3-5 | pRSFDuet-1 derivative, *E. coli* C41(DE3) *hemD* mutation 3-5; $Km^R$ | this study |
| pRSFDuet-hemD-3-6 | pRSFDuet-1 derivative, *E. coli* C41(DE3) *hemD* mutation 3-6; $Km^R$ | this study |
| pRSFDuet-hemD-3-7 | pRSFDuet-1 derivative, *E. coli* C41(DE3) | this study |

| | | |
|---|---|---|
| | *hemD* mutation 3-7; $Km^R$ | |
| pRSFDuet-hemD-4-1 | pRSFDuet-1 derivative, *E. coli* C41(DE3) *hemD* mutation 4-1; $Km^R$ | this study |
| pRSFDuet-hemD-4-2 | pRSFDuet-1 derivative, *E. coli* C41(DE3) *hemD* mutation 4-2; $Km^R$ | this study |
| pRSFDuet-hemD-5-1 | pRSFDuet-1 derivative, *E. coli* C41(DE3) *hemD* mutation 5-1; $Km^R$ | this study |
| pRSFDuet-hemD-5-2 | pRSFDuet-1 derivative, *E. coli* C41(DE3) *hemD* mutation 5-2; $Km^R$ | this study |
| pRSFDuet-hemD-6-1 | pRSFDuet-1 derivative, *E. coli* C41(DE3) *hemD* mutation 6-1; $Km^R$ | this study |
| pRSFDuet-hemD-6-2 | pRSFDuet-1 derivative, *E. coli* C41(DE3) *hemD* mutation 6-2; $Km^R$ | this study |
| pRSFDuet-hemD-6-3 | pRSFDuet-1 derivative, *E. coli* C41(DE3) *hemD* mutation 6-3; $Km^R$ | this study |
| pRSFDuet-hemD-7-1 | pRSFDuet-1 derivative, *E. coli* C41(DE3) *hemD* mutation 7-1; $Km^R$ | this study |
| pRSFDuet-hemD-7-2 | pRSFDuet-1 derivative, *E. coli* C41(DE3) *hemD* mutation 7-2; $Km^R$ | this study |
| pRSFDuet-hemD-8-1 | pRSFDuet-1 derivative, *E. coli* C41(DE3) *hemD* mutation 8-1; $Km^R$ | this study |
| pRSFDuet-hemD-8-2 | pRSFDuet-1 derivative, *E. coli* C41(DE3) *hemD* mutation 8-2; $Km^R$ | this study |
| pRSFDuet-hemD-9-1 | pRSFDuet-1 derivative, *E. coli* C41(DE3) *hemD* mutation 9-1; $Km^R$ | this study |
| pRSFDuet-hemD-9-2 | pRSFDuet-1 derivative, *E. coli* C41(DE3) *hemD* mutation 9-2; $Km^R$ | this study |
| pRSFDuet-hemC-hemD | pRSFDuet-1 derivative, *E. coli* C41(DE3) *hemC*, *hemD*; $Km^R$ | this study |
| pRSFDuet-hemC-L1-hemD | pRSFDuet-hemC-hemD derivative, *E. coli* C41(DE3) *hemC*, *hemD* connected by linker1; $Km^R$ | this study |
| pRSFDuet-hemC-L2-hemD | pRSFDuet-hemC-hemD derivative, *E. coli* C41(DE3) *hemC*, *hemD* connected by linker2; $Km^R$ | this study |
| pRSFDuet-hemC-L3-hemD | pRSFDuet-hemC-hemD derivative, *E. coli* C41(DE3) *hemC*, *hemD* connected by linker3; $Km^R$ | this study |
| pRSFDuet-CipA-hemC-hemD | pRSFDuet-hemC-hemD derivative, *E. coli* C41(DE3) *hemC*, *hemD* connected with *cipA* at the N terminal, respectively; $Km^R$ | this study |
| pRSFDuet-CipB-hemC-hemD | pRSFDuet-hemC-hemD derivative, *E. coli* C41(DE3) *hemC*, *hemD* connected with *cipB* at the N terminal, respectively; $Km^R$ | this study |

| | | |
|---|---|---|
| pRSFDuet-SAP-hemC-hemD | pRSFDuet-hemC-hemD derivative, *E. coli* C41(DE3) *hemC*, *hemD* connected with SAP(ANANARARANANARAR) at the N terminal, respectively; $Km^R$ | this study |
| pRSFDuet-YTC | pRSFDuet-hemC-hemD derivative, *E. coli* C41(DE3) *hemC* connected with spytag at the N terminal, *hem*D; $Km^R$ | this study |
| pRSFDuet-CYT | pRSFDuet-hemC-hemD derivative, *E. coli* C41(DE3) *hemC* connected with spytag at the C terminal, *hem*D; $Km^R$ | this study |
| pRSFDuet-YTD | pRSFDuet-hemC-hemD derivative, *E. coli* C41(DE3) *hemC*, *hemD* connected with spytag at the N terminal; $Km^R$ | this study |
| pRSFDuet-DYT | pRSFDuet-hemC-hemD derivative, *E. coli* C41(DE3) *hemC*, *hemD* connected with spytag at the C terminal; $Km^R$ | this study |
| pRSFDuet-Spy1-hemC-hemD | pRSFDuet-hemC-hemD derivative, *E. coli* C41(DE3) *hemC* connected with spycatcher at the N terminal, *hemD* connected with spytag at the N terminal; $Km^R$ | this study |
| pRSFDuet-Spy2-hemC-hemD | pRSFDuet-hemC-hemD derivative, *E. coli* C41(DE3) *hemC* connected with spycatcher at the N terminal, *hemD* connected with spytag at the C terminal; $Km^R$ | this study |
| pRSFDuet-Spy3-hemC-hemD | pRSFDuet-hemC-hemD derivative, *E. coli* C41(DE3) *hemC* connected with spycatcher at the C terminal, *hemD* connected with spytag at the N terminal; $Km^R$ | this study |
| pRSFDuet-Spy4-hemC-hemD | pRSFDuet-hemC-hemD derivative, *E. coli* C41(DE3) *hemC* connected with spycatcher at the C terminal, *hemD* connected with spytag at the C terminal; $Km^R$ | this study |
| pRSFDuet-Spy5-hemC-hemD | pRSFDuet-hemC-hemD derivative, *E. coli* C41(DE3) *hemC* connected with spytag at the N terminal, *hemD* connected with spycatcher at the N terminal; $Km^R$ | this study |
| pRSFDuet-Spy6-hemC-hemD | pRSFDuet-hemC-hemD derivative, *E. coli* C41(DE3) *hemC* connected with spytag at the C terminal, *hemD* connected with spycatcher at the N terminal; $Km^R$ | this study |

| | | |
|---|---|---|
| pRSFDuet-Spy7-hemC-hemD | pRSFDuet-hemC-hemD derivative, *E. coli* C41(DE3) *hemC* connected with spytag at the N terminal, *hem*D connected with spycatcher at the C terminal; $Km^R$ | this study |
| pRSFDuet-Spy8-hemC-hemD | pRSFDuet-hemC-hemD derivative, *E. coli* C41(DE3) *hemC* connected with spytag at the C terminal, *hem*D connected with spycatcher at the C terminal; $Km^R$ | this study |
| pRSFDuet-NTC | pRSFDuet-hemC-hemD derivative, *E. coli* C41(DE3) *hemC* connected with snooptag at the N terminal, *hem*D; $Km^R$ | this study |
| pRSFDuet-CNT | pRSFDuet-hemC-hemD derivative, *E. coli* C41(DE3) *hemC* connected with snooptag at the C terminal, *hem*D; $Km^R$ | this study |
| pRSFDuet-NTD | pRSFDuet-hemC-hemD derivative, *E. coli* C41(DE3) *hemC*, *hemD* connected with snooptag at the N terminal; $Km^R$ | this study |
| pRSFDuet-DNT | pRSFDuet-hemC-hemD derivative, *E. coli* C41(DE3) *hemC*, *hemD* connected with snooptag at the C terminal; $Km^R$ | this study |
| pRSFDuet-Snoop1-hemC-hemD | pRSFDuet-hemC-hemD derivative, *E. coli* C41(DE3) *hemC* connected with snoopcatcher at the N terminal, *hemD* connected with snooptag at the N terminal; $Km^R$ | this study |
| pRSFDuet-Snoop2-hemC-hemD | pRSFDuet-hemC-hemD derivative, *E. coli* C41(DE3) *hemC* connected with snoopcatcher at the N terminal, *hemD* connected with snooptag at the C terminal; $Km^R$ | this study |
| pRSFDuet-Snoop3-hemC-hemD | pRSFDuet-hemC-hemD derivative, *E. coli* C41(DE3) *hemC* connected with snoopcatcher at the C terminal, *hemD* connected with snooptag at the N terminal; $Km^R$ | this study |
| pRSFDuet-Snoop4-hemC-hemD | pRSFDuet-hemC-hemD derivative, *E. coli* C41(DE3) *hemC* connected with snoopcatcher at the C terminal, *hemD* connected with snooptag at the C terminal; $Km^R$ | this study |
| pRSFDuet-Snoop5-hemC-hemD | pRSFDuet-hemC-hemD derivative, *E. coli* C41(DE3) *hemC* connected with snooptag at the N terminal, *hemD* connected with snoopcatcher at the N terminal; $Km^R$ | this study |

| | | |
|---|---|---|
| pRSFDuet-Snoop6-hemC-hemD | pRSFDuet-hemC-hemD derivative, *E. coli* C41(DE3) *hemC* connected with snooptag at the C terminal, *hemD* connected with snoopcatcher at the N terminal; $Km^R$ | this study |
| pRSFDuet-Snoop7-hemC-hemD | pRSFDuet-hemC-hemD derivative, *E. coli* C41(DE3) *hemC* connected with snooptag at the N terminal, *hemD* connected with snoopcatcher at the C terminal; $Km^R$ | this study |
| pRSFDuet-Snoop8-hemC-hemD | pRSFDuet-hemC-hemD derivative, *E. coli* C41(DE3) *hemC* connected with snooptag at the C terminal, *hemD* connected with snoopcatcher at the C terminal; $Km^R$ | this study |
| pRSFDuet-RAC | pRSFDuet-hemC-hemD derivative, *E. coli* C41(DE3) *hemC* connected with snooptag at the N terminal, *hem*D; $Km^R$ | this study |
| pRSFDuet-CRA | pRSFDuet-hemC-hemD derivative, *E. coli* C41(DE3) *hemC* connected with snooptag at the C terminal, *hem*D; $Km^R$ | this study |
| pRSFDuet-RAD | pRSFDuet-hemC-hemD derivative, *E. coli* C41(DE3) *hemC*, *hemD* connected with snooptag at the N terminal; $Km^R$ | this study |
| pRSFDuet-DRA | pRSFDuet-hemC-hemD derivative, *E. coli* C41(DE3) *hemC*, *hemD* connected with snooptag at the C terminal; $Km^R$ | this study |
| pRSFDuet-RARD1-hemC-hemD | pRSFDuet-hemC-hemD derivative, *E. coli* C41(DE3) *hemC* connected with RIDD tag at the N terminal, *hemD* connected with RIAD tag at the N terminal; $Km^R$ | this study |
| pRSFDuet-RARD2-hemC-hemD | pRSFDuet-hemC-hemD derivative, *E. coli* C41(DE3) *hemC* connected with RIDD tag at the N terminal, *hemD* connected with RIAD tag at the C terminal; $Km^R$ | this study |
| pRSFDuet-RARD3-hemC-hemD | pRSFDuet-hemC-hemD derivative, *E. coli* C41(DE3) *hemC* connected with RIDD tag at the C terminal, *hemD* connected with RIAD tag at the N terminal; $Km^R$ | this study |
| pRSFDuet-RARD4-hemC-hemD | pRSFDuet-hemC-hemD derivative, *E. coli* C41(DE3) *hemC* connected with RIDD tag at the C terminal, *hemD* connected with RIAD tag at the C terminal; $Km^R$ | this study |
| pRSFDuet-RARD5-hemC-hemD | pRSFDuet-hemC-hemD derivative, *E. coli* C41(DE3) *hemC* connected with RIAD tag at the N terminal, *hemD* connected with RIDD tag at the N terminal; $Km^R$ | this study |

| | | |
|---|---|---|
| pRSFDuet-RARD6-hemC-hemD | pRSFDuet-hemC-hemD derivative, *E. coli* C41(DE3) *hemC* connected with RIAD tag at the C terminal, *hemD* connected with RIDD tag at the N terminal; $Km^R$ | this study |
| pRSFDuet-RARD7-hemC-hemD | pRSFDuet-hemC-hemD derivative, *E. coli* C41(DE3) *hemC* connected with RIAD tag at the N terminal, *hemD* connected with RIDD tag at the C terminal; $Km^R$ | this study |
| pRSFDuet-RARD8-hemC-hemD | pRSFDuet-hemC-hemD derivative, *E. coli* C41(DE3) *hemC* connected with RIAD tag at the C terminal, *hemD* connected with RIDD tag at the C terminal; $Km^R$ | this study |
| pETDuet-hHb | pETDuet-1 derivative; human hemoglobin α and β subunit； $Amp^R$ | this study |
| pRSFDuet-sHb | pRSFDuet-1 derivative, leghemoglobin； $Km^R$ | this study |

**Table S2.** Strains used in this study

| Strains | Description | Source |
|---|---|---|
| DH5α | *E. coli* str. K-12 $F^{-}$ *endA1 glnV44 thi-1 recA1 relA1 gyrA96 deoR nupG purB20* φ80d*lacZ*ΔM15 Δ(*lacZYA-argF*) U169 *hsdR*17($r_K^{-}m_K^{+}$)$\lambda^{-}$ | Invitrogen |
| BL21(DE3) | *E. coli* str. B $F^{-}$ *ompT gal dcm lon hsdSB*($r_B^{-}$ $m_B^{-}$ ) λ (*DE3 [lacI lacUV5-T7p07 ind1 sam7 nin5]*) [*malB*$^{+}$ ]$_{K-12}$($\lambda^{S}$ ) | Novagen |
| C41(DE3) | Derived from BL21(DE3) by selecting for resistance to OGCP overexpression | Novagen |
| C41-ΔhemA | C41(DE3) with deleting the *hemA* gene | lab storage |
| C41-D | C41(DE3) harboring plasmid pRSFDuet-hemD(WT); $Km^{R}$ | this study |
| C41-E1 | C41(DE3) harboring plasmid pACYCDuet-hemE(Ec); $Cm^{R}$ | this study |
| C41-E2 | C41(DE3) harboring plasmid pACYCDuet-hemE(Bs); $Cm^{R}$ | this study |
| C41-E3 | C41(DE3) harboring plasmid pACYCDuet-hemE(Cg); $Cm^{R}$ | this study |
| D1 | C41(DE3) harboring plasmid pRSFDuet-hemD-1-1; $Km^{R}$ | this study |
| D2 | C41(DE3) harboring plasmid pRSFDuet-hemD-1-2; $Km^{R}$ | this study |
| D3 | C41(DE3) harboring plasmid pRSFDuet-hemD-1-3; $Km^{R}$ | this study |
| D4 | C41(DE3) harboring plasmid pRSFDuet-hemD-2-1; $Km^{R}$ | this study |
| D5 | C41(DE3) harboring plasmid pRSFDuet-hemD-2-2; $Km^{R}$ | this study |
| D6 | C41(DE3) harboring plasmid pRSFDuet-hemD-3-1; $Km^{R}$ | this study |
| D7 | C41(DE3) harboring plasmid pRSFDuet-hemD-3-2; $Km^{R}$ | this study |
| D8 | C41(DE3) harboring plasmid pRSFDuet-hemD-3-3; $Km^{R}$ | this study |
| D9 | C41(DE3) harboring plasmid pRSFDuet-hemD-3-4; $Km^{R}$ | this study |
| D10 | C41(DE3) harboring plasmid pRSFDuet-hemD-3-5; $Km^{R}$ | this study |
| D11 | C41(DE3) harboring plasmid pRSFDuet-hemD-3-6; $Km^{R}$ | this study |
| D12 | C41(DE3) harboring plasmid pRSFDuet-hemD-3-7; $Km^{R}$ | this study |
| D13 | C41(DE3) harboring plasmid | this study |

| | | |
|---|---|---|
| | pRSFDuet-hemD-4-1; $Km^R$ | |
| D14 | C41(DE3) harboring plasmid pRSFDuet-hemD-4-2; $Km^R$ | this study |
| D15 | C41(DE3) harboring plasmid pRSFDuet-hemD-5-1; $Km^R$ | this study |
| D16 | C41(DE3) harboring plasmid pRSFDuet-hemD-5-2; $Km^R$ | this study |
| D17 | C41(DE3) harboring plasmid pRSFDuet-hemD-6-1; $Km^R$ | this study |
| D18 | C41(DE3) harboring plasmid pRSFDuet-hemD-6-2; $Km^R$ | this study |
| D19 | C41(DE3) harboring plasmid pRSFDuet-hemD-6-3; $Km^R$ | this study |
| D20 | C41(DE3) harboring plasmid pRSFDuet-hemD-7-1; $Km^R$ | this study |
| D21 | C41(DE3) harboring plasmid pRSFDuet-hemD-7-2; $Km^R$ | this study |
| D22 | C41(DE3) harboring plasmid pRSFDuet-hemD-8-1; $Km^R$ | this study |
| D23 | C41(DE3) harboring plasmid pRSFDuet-hemD-8-2; $Km^R$ | this study |
| D24 | C41(DE3) harboring plasmid pRSFDuet-hemD-9-1; $Km^R$ | this study |
| D25 | C41(DE3) harboring plasmid pRSFDuet-hemD-9-2; $Km^R$ | this study |
| R1 | C41(DE3) harboring plasmid pRSFDuet-hemC-hemD; $Km^R$ | this study |
| L1 | C41(DE3) harboring plasmid pRSFDuet-hemC-L1-hemD; $Km^R$ | this study |
| L2 | C41(DE3) harboring plasmid pRSFDuet-hemC-L2-hemD; $Km^R$ | this study |
| L3 | C41(DE3) harboring plasmid pRSFDuet-hemC-L3-hemD; $Km^R$ | this study |
| H1 | C41(DE3) harboring plasmid pRSFDuet-CipA-hemC-hemD; $Km^R$ | this study |
| H2 | C41(DE3) harboring plasmid pRSFDuet-CipB-hemC-hemD; $Km^R$ | this study |
| H3 | C41(DE3) harboring plasmid pRSFDuet-SAP-hemC-hemD; $Km^R$ | this study |
| Y1 | C41(DE3) harboring plasmid pRSFDuet-Spy1-hemC-hemD; $Km^R$ | this study |
| Y2 | C41(DE3) harboring plasmid pRSFDuet-Spy2-hemC-hemD; $Km^R$ | this study |
| Y3 | C41(DE3) harboring plasmid | this study |

| | | |
|---|---|---|
| | pRSFDuet-Spy3-hemC-hemD; $Km^R$ | |
| Y4 | C41(DE3) harboring plasmid pRSFDuet-Spy4-hemC-hemD; $Km^R$ | this study |
| Y5 | C41(DE3) harboring plasmid pRSFDuet-Spy5-hemC-hemD; $Km^R$ | this study |
| Y6 | C41(DE3) harboring plasmid pRSFDuet-Spy6-hemC-hemD; $Km^R$ | this study |
| Y7 | C41(DE3) harboring plasmid pRSFDuet-Spy7-hemC-hemD; $Km^R$ | this study |
| Y8 | C41(DE3) harboring plasmid pRSFDuet-Spy8-hemC-hemD; $Km^R$ | this study |
| N1 | C41(DE3) harboring plasmid pRSFDuet-Snoop1-hemC-hemD; $Km^R$ | this study |
| N2 | C41(DE3) harboring plasmid pRSFDuet-Snoop2-hemC-hemD; $Km^R$ | this study |
| N3 | C41(DE3) harboring plasmid pRSFDuet-Snoop3-hemC-hemD; $Km^R$ | this study |
| N4 | C41(DE3) harboring plasmid pRSFDuet-Snoop4-hemC-hemD; $Km^R$ | this study |
| N5 | C41(DE3) harboring plasmid pRSFDuet-Snoop5-hemC-hemD; $Km^R$ | this study |
| N6 | C41(DE3) harboring plasmid pRSFDuet-Snoop6-hemC-hemD; $Km^R$ | this study |
| N7 | C41(DE3) harboring plasmid pRSFDuet-Snoop7-hemC-hemD; $Km^R$ | this study |
| N8 | C41(DE3) harboring plasmid pRSFDuet-Snoop8-hemC-hemD; $Km^R$ | this study |
| A1 | C41(DE3) harboring plasmid pRSFDuet-RARD1-hemC-hemD; $Km^R$ | this study |
| A2 | C41(DE3) harboring plasmid pRSFDuet-RARD2-hemC-hemD; $Km^R$ | this study |
| A3 | C41(DE3) harboring plasmid pRSFDuet-RARD3-hemC-hemD; $Km^R$ | this study |
| A4 | C41(DE3) harboring plasmid pRSFDuet-RARD4-hemC-hemD; $Km^R$ | this study |
| A5 | C41(DE3) harboring plasmid pRSFDuet-RARD5-hemC-hemD; $Km^R$ | this study |
| A6 | C41(DE3) harboring plasmid pRSFDuet-RARD6-hemC-hemD; $Km^R$ | this study |
| A7 | C41(DE3) harboring plasmid pRSFDuet-RARD7-hemC-hemD; $Km^R$ | this study |
| A8 | C41(DE3) harboring plasmid pRSFDuet-RARD8-hemC-hemD; $Km^R$ | this study |
| GD1 | C41(DE3) with constitutive promoter $P_{J23100}$ added | this study |

| | | |
|---|---|---|
| | for *hemD* gene | |
| GD2 | C41(DE3) with constitutive promoter $P_{J23104}$ added for *hemD* gene | this study |
| GD3 | C41(DE3) with constitutive promoter $P_{J23106}$ added for *hemD* gene | this study |
| GD4 | C41(DE3) with constitutive promoter $P_{J23114}$ added for *hemD* gene | this study |
| GD5 | C41(DE3) with constitutive promoter $P_{J23117}$ added for *hemD* gene | this study |
| GA1 | C41(DE3) derivative, *hemC*, *hemD* connected by linker3 (GGGGSGGGGSGGGGS) | this study |
| GA2 | C41(DE3) derivative, *hemC* connected with spycatcher at the C terminal, *hemD* connected with spytag at the N terminal | this study |
| GA3 | C41(DE3) derivative, *hemC* connected with RIAD tag at the C terminal, *hemD* connected with RIDD tag at the N terminal | this study |
| GA4 | C41(DE3) derivative, *hemC* connected with spycatcher at the C terminal, *hemD* connected with spytag at the N terminal and expressed through constitutive promoter $P_{J23104}$ | this study |
| GA5 | C41(DE3) derivative, *hemC* connected with RIAD tag at the C terminal, *hemD* connected with RIDD tag at the N terminal and expressed through constitutive promoter $P_{J23104}$ | this study |
| C41-ΔtolC | C41(DE3) with deleting *tolC* gene | this study |
| C41- Δ ybhRS | C41(DE3) with deleting *ybhRS* gene | this study |
| C41-ΔyddA | C41(DE3) with deleting *yddA* gene | this study |
| C41-ΔyojI | C41(DE3) with deleting *yojI* gene | this study |
| C41-ΔmacAB | C41(DE3) with deleting *macAB* gene | this study |
| C41-ΔtolC- Δ ybhRS | C41(DE3) with deleting *tolC* and *ybhRS* genes | this study |
| C41-ΔtolC-ΔyddA | C41(DE3) with deleting *tolC* and *yddA* genes | this study |
| C41-ΔtolC-ΔyojI | C41(DE3) with deleting *tolC* and *yojI* genes | this study |
| C41-ΔtolC-ΔmacAB | C41(DE3) with deleting *tolC* and *macAB* genes | this study |
| GH0 | C41-ΔtolC with the promoter of *hemH* replaced by $P_{J23100}$ promoter and RBS (ATAAAAGGAGGAAAATAT) | this study |
| GH1 | GH0 derivative, *hemC* connected with RIAD tag at the C terminal, *hemD* connected with RIDD tag at the N terminal and expressed through constitutive promoter PJ23104 | this study |
| GH2 | GH1 derivative, $P_{J23100}$ promoter and RBS (ATAAAAGGAGGAAAATAT) added before | this study |

| | | |
|---|---|---|
| | *hemE* gene | |
| GH3 | GH2 with deleting the *yfeX* gene | this study |
| HEME1 | GH3 derivative, the fragment $P_{J23100}$-*hemA-hemL* integrated into the *cheW* locus | this study |
| HEME2 | GH3 derivative, the fragment $P_{J23100}$-*hemA-hemL* integrated into the *cheW* and *yciQ* locus | this study |
| HEME3 | GH3 derivative, the fragment $P_{J23100}$-hemA-hemL integrated into the *cheW*, *yciQ*, and *mbhA* locus | this study |
| C41-hHb | C41(DE3) harboring plasmid pETDuet-hHb; $Amp^R$ | this study |
| HEME2-hHb | HEME2 harboring plasmid pETDuet-hHb; $Amp^R$ | this study |
| C41-sHb | C41(DE3) harboring plasmid pRSFDuet-sHb; $Km^R$ | this study |
| HEME2-sHb | HEME2 harboring plasmid pRSFDuet-sHb; $Km^R$ | this study |

**Table S3.** Primers used in this study

| Primers | Sequence (5'-3') |
|---|---|
| hemD-F | aaggagatataccatgggcagtatcctggtcacccg |
| hemD-R | ccgagctcgaattcggatccttattgtaatgcccgtaaaa |
| 1-1 I32R-F | cattttccactgCGCgagttttctccgggtcgacaattacca |
| 1-1 I32R-R | ggagaaaactcGCGcagtggaaaatgccaggccacc |
| 1-2 I32H-F | cattttccactgCATgagttttctccgggtcgacaattacca |
| 1-2 I32H-R | ggagaaaactcATGcagtggaaaatgccaggccacc |
| 1-3 I32K-F | attttccactgAAAgagttttctccgggtcgacaattacca |
| 1-3-I32K-R | ggagaaaactcTTTcagtggaaaatgccaggccacc |
| 2-1 F34R-F | cactgattgagCGCtctccgggtcgacaattaccacaac |
| 2-1 F34R-R | acccggagaGCGctcaatcagtggaaaatgccaggc |
| 2-2 F34K-R | cccggagaTTTctcaatcagtggaaaatgccaggcc |
| 2-2-F34K-F | cactgattgagAAAtctccgggtcgacaattaccacaact |
| 3-1 L59R-F | ttgtttgccCGCtcgcaacacgcggttgc |
| 3-1 L59R-R | gtgttgcgaGCGggcaaacaacagatcgctctcc |
| 3-2 L59C-F | tgtttgccTGCtcgcaacacgcggttgc |
| 3-2 L59C-R | gtgttgcgaGCAggcaaacaacagatcgctctccC |
| 3-3 L59G-F | gttgtttgccGGCtcgcaacacgcggttgc |
| 3-3 L59G-R | gtgttgcgaGCCggcaaacaacagatcgctctccc |
| 3-4 L59H-F | ttgtttgccCATtcgcaacacgcggttgc |
| 3-4 L59H-R | gtgttgcgaATGggcaaacaacagatcgctctcc |
| 3-5 L59K-F | gttgtttgccAAAtcgcaacacgcggttgc |
| 3-5 L59K-R | cgtgttgcgaTTTggcaaacaacagatcgctctccc |
| 3-6 L59P-F | ttgtttgccCCGtcgcaacacgcggttgc |
| 3-6 L59P-R | cgtgttgcgaCGGggcaaacaacagatcgctctcc |
| 3-7 L59V-F | gttgtttgccGTGtcgcaacacgcggttgc |
| 3-7 L59V-R | cgtgttgcgaCACggcaaacaacagatcgctctccc |
| 4-1 Q61R-F | ccctctcgCGCcacgcggttgcttttgcc |
| 4-1 Q61R-R | accgcgtgGCGcgagagggcaaacaacagat |
| 4-2 Q61K-F | ccctctcgAAAcacgcggttgcttttgcc |
| 4-2 Q61K-R | accgcgtgTTTcgagagggcaaacaacaga |
| 5-1 H62R-F | tctcgcaaCGCgcggttgcttttgccc |
| 5-1 H62R-R | caaccgcGCGttgcgagagggcaaacaacag |
| 5-2 H62K-F | cctctcgcaaAAAgcggttgcttttgcccaatcac |
| 5-2 H62K-R | gcaaccgcTTTttgcgagagggcaaacaacagatc |
| 6-1 N135R-F | attacgtggcCGCggcggtcgtgagctaattggg |
| 6-1 N135R-R | cgaccgccGCGgccacgtaatatcagcgcacgttt |
| 6-2 N135H-F | attacgtggcCATggcggtcgtgagctaattgg |
| 6-2 N135H-R | gaccgccATGgccacgtaatatcagcgcacg |
| 6-3 N135K-F | acgtggcAAAggcggtcgtgagctaattgg |
| 6-3 N135K-R | cgaccgccTTTgccacgtaatatcagcgcacg |
| 7-1 G187R-F | ttgttaccagcCGCgaaatgttgcagcaactctggtcg |
| 7-1 G187R-R | tgcaacatttcGCGgctggtaacaacgaccgtcgt |

| | |
|---|---|
| 7-2 G187K-F | ttgttaccagcAAAgaaatgttgcagcaactctggtcg |
| 7-2 G187K-R | tgcaacatttcTTTgctggtaacaacgaccgtcgt |
| 8-1 M189H-F | agcggtgaaCATttgcagcaactctggtcgctg |
| 8-1 M189H-R | gttgctgcaaATGttcaccgctggtaacaacgacc |
| 8-2 M189K-F | gcggtgaaAAAttgcagcaactctggtcgctg |
| 8-2 M189K-R | gttgctgcaaTTTttcaccgctggtaacaacgacc |
| 9-1 L218R-F | agtgagcgtCGCgcgaaactcgcccgggaa |
| 9-1 L218R-R | cgagtttcgcGCGacgctcactgacgaccaatagtc |
| 9-2 L218K-F | agtgagcgtAAAgcgaaactcgcccgggaa |
| 9-2 L218K-R | cgagtttcgcTTTacgctcactgacgaccaatagtc |
| hemE (Ec)-F | ataaggagatataccatggGCaccgaacttaaaaacgat |
| hemE (Ec)-R | gagctcgaattcggatccttagcggtgatattgttcag |
| hemE (Bs)-F | ctttaataaggagatataccatggGCagtaaacgagaaacgtttaa |
| hemE (Bs)-R | ccgagctcgaattcggatccttaggaatattgacccattt |
| hemE (Cg)-F | taaggagatataccatggGCTCTGCTCTTACTATTCCAGC |
| hemE (Cg)-R | cgagctcgaattcggatccTTAAGAATGAATGATGGAGA |
| J23100-hemE-F | ctcagtcctaggtacagtgctagcATAAAAGGAGGAAAATATatgaccgaacttaaaaacgatcg |
| J23100-hemE-R | gcactgtacctaggactgagctagccGTCAAatttcctaatgcaggagt |
| hemC-F | aaggagatataccatgggcttagacaatgttttaaga |
| hemC-R | catATATTTTCCTCCTTTTATtcatgccggagcgtctccgt |
| hemD-F2 | ccggcatgaATAAAAGGAGGAAAATATatgagtatcctggtcacccgc |
| L1-F | gctccggcaGGTGGTGGTAGCatgag |
| L1-R | GCTACCACCACCtgccggagcgtctc |
| L2-F | TGGTAGCGGTGGTGGTAGCGGTatgagtatcctggtcac |
| L2-R | CTACCACCACCGCTACCACCtgccggagcgtctc |
| L3-F | GGTAGCGGTGGTGGTGGTAGCGGTGGTGGTGGTACGatgagtatcctggtcac |
| L3-R | CACCACCACCGCTACCACCACCACCtgccggagcgtctc |
| cipA-C-F | actttaataaggagatataccATGATTAATGATATGCACCC |
| cipA-C-R | aacattgtctaagcccatCATGGAGATCTCGACGCA |
| cipA-D-F | tgaATAAAAGGAGGAAAATATATGATTAATGATATGCACCC |
| cipA-D-R | ggtgaccaggatactcatCATGGAGATCTCGACGCA |
| hemC-cipA-F | ATGatgggcttagacaatgttttaagaattgcca |
| hemC-cipA-R | catATATTTTCCTCCTTTTATtcatgccggagcgtctccgt |
| RSF-cipA-F | AGATCTCCATGatgagtatcctggtcacccgcc |
| RSF-cipA-R | ATCATTAATCATggtatatctccttattaaagttaaacaaaattatttctac |
| cipB-C-F | ttaataaggagatataccATGATTATCAAGAAAGATATTCTATTA |
| cipB-C-R | aagcccatTGCGCTTGCACCGTTGCTTGCGCTTGCGATTTCCACACCCAC |
| cipB-D-F | gaATAAAAGGAGGAAAATATATGATTATCAAGAAAGATATTCTATTA |
| cipB-D-R | ggatactcatTGCGCTTGCACCGTTGCTTGCGCTTGCGATCTCT |

| | |
|---|---|
| | ACGCCCACA |
| hemC-cipB-F | AAGCGCAAGCAACGGTGCAAGCGCAatgggcttagacaatgtt |
| hemC-cipB-R | TAATCATATATTTTCCTCCTTTTATtcatgccggagcgtctccgtta |
| RSF-cipB-F | GCGCAAGCAACGGTGCAAGCGCAatgagtatcctggtcac |
| RSF-cipB-R | CATggtatatctccttattaaagttaaacaaaattatttctacagggg |
| SAP-F | GCTAATGCGAACGCTCGCGCACGTggcttagacaatgttt |
| SAP-R | GCGTTCGCATTAGCCCTTGCTCTGGCATTCGCATTAGCCA<br>TATATTTTCCTCCTTTTA |
| RSF-SAP-F | AAGGGCTAATGCGAACGCTCGCGCACGTagtatcctggtcaccc |
| RSF-SAP-R | GAGCGTTCGCATTAGCCCTTGCTCTGGCATTCGCATTAGC<br>CATggtatatctccttat |
| DNT-F | GGTGGTGGTAGCGCTAGTAAACTAGGAGATATTGAATTT<br>ATAAAGGTGAACAAATAAggatccgaattcga |
| DNT-R | TTTACTAGCGCTACCACCACCACCGCTACCACCACCACCtt<br>gtaatgcccgtaaaa |
| NTD-F | TATTGAATTTATAAAGGTGAACAAAGGTGGTGGTGGTAG<br>CGGTGGTGGTGGTAGCatgagtatcctggtca |
| NTD-R | CTTTATAAATTCAATATCTCCTAGTTTACTAGCcatATATTT<br>TCCTCCTTTTATtcatgccggagcgtct |
| CNT-F | TGGTAGCGCTAGTAAACTAGGAGATATTGAATTTATAAA<br>GGTGAACAAATAAATAAAAGGAGGAAAATA |
| CNT-R | CTAGTTTACTAGCGCTACCACCACCACCGCTACCACCACC<br>ACCtgccggagcgtctccgtt |
| NTC-F | TTATAAAGGTGAACAAAGGTGGTGGTGGTAGCGGTGGTG<br>GTGGTAGCatgttagacaatgttttaa |
| NTC-R | CCACCTTTGTTCACCTTTATAAATTCAATATCTCCTAGTTT<br>ACTAGCcatggtatatctccttatta |
| CYT-F | TGGTAGCGCTCACATAGTAATGGTTGATGCATATAAACC<br>GACCAAGTAAATAAAAGGAGGAAAATATa |
| CYT-R | TTACTATGTGAGCGCTACCACCACCACCGCTACCACCAC<br>CACCtgccggagcgtctccgtt |
| DYT-F | GGTGGTAGCGCTCACATAGTAATGGTTGATGCATATAAA<br>CCGACCAAGTAAggatccgaattcga |
| DYT-R | TATGTGAGCGCTACCACCACCACCGCTACCACCACCACCt<br>tgtaatgcccgtaaaa |
| YTD-F | TTGATGCATATAAACCGACCAAGGGTGGTGGTGGTAGCG<br>GTGGTGGTGGTAGCatgagtatcctggtca |
| YTD-R | GGTTTATATGCATCAACCATTACTATGTGAGCcatATATTT<br>TCCTCCTTTTATtcatgccggagcgtct |
| YTC-F | AAACCGACCAAGGGTGGTGGTGGTAGCGGTGGTGGTGGT<br>AGCatgttagacaatgttttaa |
| YTC-R | CCACCACCCTTGGTCGGTTTATATGCATCAACCATTACTA<br>TGTGAGCcatggtatatctccttatta |
| YC-F | taataaggagatataccatgGCTATGGTTGATACCCTGTCCGGCC |

| | |
|---|---|
| YC-R | CTCCACCTGAACCTCCACCACCGATGTGGGCGTCACCTTT |
| RSF-YC-F | GGTGGAGGTTCAGGTGGAGGCGGAAGTatgttagacaatgttttaa |
| RSF-YC-R | TCAACCATAGCcatggtatatctccttattaaagttaaacaa |
| CY-F | ctccggcaGGTGGAGGCGGAAGTGGCGG |
| CY-R | ATATTTTCCTCCTTTTATTTAGATGTGGGCGTCACCTT |
| RSF-CY-F | TGACGCCCACATCTAAATAAAAGGAGGAAAATATatg |
| RSF-CY-R | GCCTCCACCtgccggagcgtctccgttata |
| YD-F | ATAAAAGGAGGAAAATATatgGCTATGGTTGATACCCTGTCCGG |
| YD-R | TCCACCTGAACCTCCACCACCGATGTGGGCGTCACC |
| RSF-YD-F | TGGAGGTTCAGGTGGAGGCGGAAGTatgagtatcctggtca |
| RSF-YD-R | ACAGGGTATCAACCATAGCcatATATTTTCCTCCTTTTATT |
| DY-F | cattacaaGGTGGAGGCGGAAGTGGCGGTGG |
| DY-R | agctcgaattcggatccTTAGATGTGGGCGTCACCT |
| RSF-DY/N-F | ggatccgaattcgagctcggcgc |
| RSF-DY-R | GCCTCCACCttgtaatgcccgtaaaagcgcatcg |
| NC-F | taataaggagatataccatgGCGAGCAAACCGCTGCGCGG |
| NC-R | TCCACCTGAACCTCCACCACCCTTAGGCGGAATCGGCTCAT |
| RSF-NC-F | GGTGGAGGTTCAGGTGGAGGCGGAAGTatgttagacaatgttttaag |
| RSF-NC-R | GGTTTGCTCGCcatggtatatctccttattaaagttaaa |
| C/DN-F | GGTGGAGGCGGAAGTGGTGGTGGCGGTTCTGC |
| CN-R | CCTCCTTTTATTTACTTAGGCGGAATCGGCTCATTGGTGAT |
| RSF-CN-F | CAATGAGCCGATTCCGCCTAAGTAAATAAAAGGAGGAAAATATatg |
| RSF-CN-R | CCACCACTTCCGCCTCCACCtgccggagcgtctccgttat |
| ND-F | TAAAAGGAGGAAAATATatgGCGAGCAAACCGCTGCGCGG |
| ND-R | CACCTGAACCTCCACCACCCTTAGGCGGAATCGGCTCAT |
| RSF-ND-F | GTGGTGGAGGTTCAGGTGGAGGCGGAAGTatgagtatcctggtcac |
| RSF-ND-R | AGCGGTTTGCTCGCcatATATTTTCCTCCTTTTAT |
| DN-R | gagctcgaattcggatccTTACTTAGGCGGAATCGGCT |
| RSF-DN-R | ACCACTTCCGCCTCCACCttgtaatgcccgtaaaagc |
| D-RA-F | GTATGCAAATCAGCTGGCAGATCAGATTATCAAAGAAGCAACCGAAGGTTGCTAAggatccgaattcga |
| D-RA-R | CAGCTGATTTGCATACTGTTCCAGGCTACCACCACCACCGCTACCACCACCACCG |
| RA-D-F | ATCAGATTATCAAAGAAGCAACCGAAGGTTGCGGTGGTGGTGGTAGCGGTGGTGGTGGTAGCGG |
| RA-D-R | GCTTCTTTGATAATCTGATCTGCCAGCTGATTTGCATACTGTTCCAGcatATATTTTCCTCCTTTTAT |
| C-RA-F | AATCAGCTGGCAGATCAGATTATCAAAGAAGCAACCGAA |

| | |
|---|---|
| | GGTTGCTAAATAAAAGGAGGAAAATAT |
| C-RA-R | ATCTGATCTGCCAGCTGATTTGCATACTGTTCCAGGCTACCACCACCACCGCTACCACCACCACCGCTA |
| RA-C-F | ATCAGATTATCAAAGAAGCAACCGAAGGTTGCGGTGGTGGTGGTAGCGGTGGTGGTGGTAGCGG |
| RA-C-R | CTTCTTTGATAATCTGATCTGCCAGCTGATTTGCATACTGTTCCAGGCCCATggtatatctccttatt |
| RDC-F | taaggagatataccATGggcAGCCTGCGTGAATGTGAACT |
| RDC-R | aaaacattgtctaacatACCACAACCACCACCACCTG |
| RSF-RDC-F | atgttagacaatgttttaagaattgccacacgccaaag |
| RSF-RDC-R | GGCTgccCATggtatatctccttattaaag |
| CRD-F | TGGTGGTGGTTCAGGTGGTGGTGGTTGTGGTAGCCTGCGTGAATGTGAACT |
| CRD-R | catATATTTTCCTCCTTTTATttaTTTGGCTTCTTCTTTTTCCAGACG |
| RSF-CRD-F | CGTCTGGAAAAAGAAGAAGCCAAAtaaATAAAAGGAGGAAAATATatg |
| RSF-CRD-R | ACCTGAACCACCACCACCTGAACCACCACCACCtgccggagcgtctccgtta |
| RDD-F | TAAATAAAAGGAGGAAAATATATGAGCCTGCGTGAATGTGA |
| RDD-R | gtgaccaggatactcatACCACAACCACCACCACCTGAA |
| RSF-RDD-F | atgagtatcctggtcacccgcccgtc |
| RSF-RDD-R | ACATTCACGCAGGCTCATATATTTTCCTCCTTTTATTTA |
| DRD-F | GGTGGTGGTTCAGGTGGTGGTGGTTGTGGTAGCCTGCGTGAATGTGAA |
| DRD-R | gcgccgagctcgaattcggatccttaTTTGGCTTCTTCTTTTTCCA |
| RSF-DRD-F | ggatccgaattcgagctcggcgcgcct |
| RSF-DRD-R | CACCACCTGAACCACCACCACCTGAACCACCACCACCttgtaatgcccgtaaaa |
| sgRNA-tolC-F | taatactagtACGTAATAACCTTGATAACGgttttagagctagaaatagc |
| sgRNA-tolC-R | gctctaaaacCGTTATCAAGGTTATTACGTactagtattatacctaggac |
| Up-tolC-F | atcatcccggcaaccatctccag |
| Up-tolC-R | TCCTTGTGGTGAAGCAGTATTTAGCGC |
| Down-tolC-F | TGACGACGACGGGGCTTCGG |
| Down-tolC-R | CTGGATTGCTGGGCCTGCGCCTG |
| sgRNA-msbA-F | taatactagtACGACAAAGATCTCTCTACGgttttagagctagaaatagc |
| sgRNA-msbA-R | aaaacCGTAGAGAGATCTTTGTCGTactagtattatacctaggac |
| sgRNA-macAB-F | actagtGCGCGGGCATTGATGAACGGgttttagagctagaaata |
| sgRNA-macAB-R | ATCAATGCCCGCGCactagtattatacctaggactg |
| sgRNA-YddA- | actagtACCGATCACAATGAGCACAAgttttagagctagaaata |

| | |
|---|---|
| F | |
| sgRNA-YddA-R | aaacTTGTGCTCATTGTGATCGGTactagtattatacctagga |
| sgRNA-YojI-F | tataatactagtACACCTGATAAAACTCACGAgttttagagctagaaat |
| sgRNA-YojI-R | **tagctctaaaacTCGTGAGTTTTATCAGGTGTactagtattatacctag gac** |
| sgRNA-YbhRS-F | actagtAAGGTGGCGACAATTAACGCgttttagagctagaaata |
| sgRNA-YbhRS-R | ATTGTCGCCACCTTactagtattatacctaggactgag |
| Down-MacAB-F | TTTTTGAGATAAAAATGCCAGCCGATCGGGCT |
| Down-MacAB-R | GACATCTGTCACATTCCTGTCAATAGCGTTAACTGC |
| Up-MacAB-F | TCTTCCGCCGACATACATCCGCGGA |
| Up-MacAB-R | ATCGGCTGGCATTTTTATCTCAAAAAAAATATTCTGAATA CTCCAT |
| Down-MsbA-F | TCGAAAAAATCTGGTCTGGTGAATCCCC |
| Down-MsbA-R | ACGCACACCATCAATAACGACAATTTCCA |
| Up-MsbA-F | TGCGTTTCAGCGCGCACTGG |
| Up-MsbA-R | ACCAGACCAGATTTTTTCGATCAAAAAACCAGCATTTGTT |
| Down-YbhRS-F | TGCTATGCTCCTTATCCCAGTCGTG |
| Down-YbhRS-R | ATTTTCGCTCTACGGCAACCTGAC |
| Up-YbhRS-F | TTCCTGTCTTTGCCTCTTATCTCATTGAAATAGTGT |
| Up-YbhRS-R | GGGATAAGGAGCATAGCACTTCTTATCTTTCGCCAG |
| Down-YddA-F | TAGCGAAAATTGATTGTGCCATCCAATGATTA |
| Down-YddA-R | CAATGTGCATATAAACCTATCTGCAATGGCG |
| Up-YddA-F | CAAAACTGTCATAAAGCGTCACATTGTCCAGT |
| Up-YddA-R | GGCACAATCAATTTTCGCTAAAACAATAAAAGCCCTGCT G |
| Down-YojI-F | GCAGAAAACCCGGACAATGAATTACAGCC |
| Down-YojI-R | ATCTCTATTCGCCCATTGATCCGCAAAC |
| Up-YojI-R | TTGTCCGGGTTTTCTGCTATTACGACGCTGACCT |
| Up-YojI-F | TTTGCGGGGTGTAGTTCAGTTCCATCAGTG |
| sgRNA-hemD-F | ggtataatactagtGCTCACTAACTCTTCTCCAGgttttagagctagaaatagc |
| sgRNA-hemD-R | agctctaaaacCTGGAGAAGAGTTAGTGAGCactagtattatacctaggac |
| Up-hemD-F | GCTGCAGCGGTCGTGACGTC |
| Up-114-hemD-R | tttatggctagctcagtcctaggtacaatgctagcATAAAAGGAGGAAAATAT atgagtatcctggtcac |
| Up-117-hemD-R | ttgacagctagctcagtcctagggattgtgctagcATAAAAGGAGGAAAATA Tatgagtatcctggtcacc |

| | |
|---|---|
| Up-100-hemD-R | ACggctagctcagtcctaggtacagtgctagcATAAAAGGAGGAAAATATatgagtatcctggtcac |
| Up-106-hemD-R | ACGggctagctcagtcctaggtatagtgctagcATAAAAGGAGGAAAATATatgagtatcctggtcacc |
| Up-104-hemD-R | TTGACagctagctcagtcctaggtattgtgctagcATAAAAGGAGGAAAATATatgagtatcctggtcaccc |
| Down-114-hemD-F | aggactgagctagccataaatcatgccggagcgtc |
| Down-117-hemD-F | ctgagctagctgtcaatcatgccggagcgtc |
| Down-100-hemD-F | ggactgagctagccGTCAAtcatgccggagcgtctc |
| Down-106-hemD-F | ctaggactgagctagccCGTAAtcatgccggagcgtctc |
| Down-104-hemD-F | gactgagctagctGTCAAtcatgccggagcgtctc |
| Down-hemD-R | tgcgaggcaacgtcggcactc |
| 104-RA-hemC-F | caatacctaggactgagctagctGTCAATTAGCAACCTTCGGTTGCTT |
| hemD-F2 | GGCTTCCTGTTATGAGAGttattgtaatgcccgta |
| 104-RD-R | tcagtcctaggtattgtgctagcATAAAAGGAGGAAAATATATGAGCC |
| 104-SPY-hemC-F | atacctaggactgagctagctGTCAATTAGATGTGGGCGTCACCTT |
| 104-SPY-hemD-R | gctagctcagtcctaggtattgtgctagcATAAAAGGAGGAAAATATatgGCTCACATAG |
| sgRNA-yfeX-F | ggtataatactagtTGACGGTACAGAAAACCCGGgttttagagctagaaatagc |
| sgRNA-yfeX-R | agctctaaaacCCGGGTTTTCTGTACCGTCAactagtattatacctaggac |
| sgRNA-hemE-F | ggtataatactagtGCTGGGTTACGTGATGAACGgttttagagctagaaatagc |
| sgRNA-hemE-R | agctctaaaacCGTTCATCACGTAACCCAGCactagtattatacctaggac |
| UP-yfeX-F | ggggcgattattcccggcctgct |
| UP-yfeX-R | ctgagctagccGTCAAgcgtttctgattcattgat |
| Down-yfeX-F | CGCTAAtgttcctcctgaaaataagaatgcca |
| Down-yfeX-R | caagccattgctgatgcgcttgatg |
| UP-hemE-F | ACCGATCGCATAAATACTGTGGTTACTGCGG |
| UP-100-hemE-R | cactgtacctaggactgagctagccGTCAAtttggctgttccttagtgcg |
| 100-hemE-F | ctcagtcctaggtacagtgctagcATAAAAGGAGGAAAATATATGACCGAACTTAAAAACGATCG |
| Down-hemE-R | CGCCAGACCCAGGCCAGCT |
| sgRNA-hemH-F | ggtataatactagtGATTGCCGAGCAAAACCGTGgttttagagctagaaatagc |

| | |
|---|---|
| sgRNA-hemH-R | agctctaaaacCACGGTTTTGCTCGGCAATCactagtattatacctaggac |
| UP-hemH-F | gccgtctggtcgtgtttatcacgtt |
| UP-hemH-R | ctgtacctaggactgagctagccGTCAAatcagaaaaaggaatagc |
| 100-hemH-F | ctcagtcctaggtacagtgctagcATAAAAGGAGGAAAATATATGACCGAACTTAAAAACGATCG |
| Down-hemH-R | caaatcaatatgtcccgcattggctt |
| sgRNA-CheW-F | ggtataatactagtAAAGGCGTCACGAATCTGCGgttttagagctagaaatagc |
| sgRNA-CheW-R | agctctaaaacCGCAGATTCGTGACGCCTTTactagtattatacctaggac |
| UP-CheW-F | gtagcgaccatttcaggtaacggtgcc |
| UP-CheW-R | ttctcccgatttcctcaattgaaatgaaccc |
| DOWN-CheW-F | attgttaccttttactcattcaggcggagg |
| DOWN-CheW-R | ggtactggcactacgatccgcattttactg |
| hemAL-CheW-F | aattgaggaaatcgggagaaTTGACggctagctcagt |
| hemAL-CheW-R | atgagtaaaaaggtaacaattcacaacttcgcaaacaccc |
| hemA-F | aggtacagtgctagcATAAAAGGAGGAAAATATatgaccctttagcactc |
| hemA-R | ATATTTTCCTCCTTTTATctactccagcccgaggct |
| hemL-F | gagtagATAAAAGGAGGAAAATATatgagtaagtctgaaaatcttt |
| hemL-R | ctagtttaattaactcgagtcacaacttcgcaaacacccg |
| sgRNA-mbhA-F | ggtataatactagtAATCGCAATATGTTCGAACGgttttagagctagaaatagc |
| sgRNA-mbhA-R | agctctaaaacCGTTCGAACATATTGCGATTactagtattatacctaggac |
| sgRNA-yciQ-F | ggtataatactagtATAACCGGTCAGGAAAACAGgttttagagctagaaatagc |
| sgRNA-yciQ-R | agctctaaaacCTGTTTTCCTGACCGGTTATactagtattatacctaggac |
| Down-mbhA-F | atgctgaatctttacgcatttctcaaactctga |
| Down-mbhA-R | tgccggaacttctgccggcgtaatca |
| UP-mbhA-F | agcggctgtttgctcatccgc |
| UP-mbhA-R | ttactgacgctgttgacaatcaactttatcgtc |
| hemAL-mbhA-F | attgtcaacagcgtcagtaaTTGACggctagctcagtc |
| hemAL-mbhA-R | aatgcgtaaagattcagcattcacaacttcgcaaacaccc |
| Down-yciQ-F | ttaagctgatgttaatcgaaacaagctttaaccat |
| Down-yciQ-R | tttggcagacggtcaaacaccgtcgg |
| UP-yciQ-F | cgcgatctttctgaactctcgacctatt |
| UP-yciQ-R | agtcttccatccatggaacgcaatttgtt |

| | |
|---|---|
| hemAL-yciQ-F | ttccatggatggaagactTTGACggctagctcagtc |
| hemAL-yciQ-R | ttcgattaacatcagcttaatcacaacttcgcaaacaccc |

**Table S4.** The result of molecular docking for HemD and HMB

| Conformation | -Codocker energy | -Codocker interaction energy | Distance between Y158 and C20 of HMB (Å) |
|---|---|---|---|
| 1 | 133.534 | 120.901 | 11.303 |
| 2 | 133.019 | 121.929 | 11.175 |
| 3 | 132.542 | 122.275 | 11.236 |
| 4 | 132.404 | 122.13 | 6.213 |
| 5 | 131.507 | 124.056 | 5.519 |
| 6 | 131.498 | 121.313 | 12.233 |
| 7 | 131.454 | 125.232 | 10.390 |
| 8 | 130.384 | 125.769 | 12.759 |
| 9 | 129.941 | 120.251 | 10.983 |
| 10 | 129.205 | 121.461 | 10.421 |

**Table S5.** The mutations of HemD predicted by simulate saturation mutagenesis of residues within a distance of 5 Å around the small molecule

| Number | Mutations | Mutation energy (kcal/mol) |
|---|---|---|
| 1-1 | ILE32>ARG | -2.02 |
| 1-2 | ILE32>HIS | -1.18 |
| 1-3 | ILE32>LYS | -2.34 |
| 2-1 | PHE34>ARG | -1.77 |
| 2-2 | PHE34>LYS | -1.88 |
| 3-1 | LEU59>ARG | -1.39 |
| 3-2 | LEU59>CYS | -0.61 |
| 3-3 | LEU59>GLY | -0.54 |
| 3-4 | LEU59>HIS | -1.17 |
| 3-5 | LEU59>LYS | -3.14 |
| 3-6 | LEU59>PRO | -0.61 |
| 3-7 | LEU59>VAL | -0.62 |
| 4-1 | GLN61>ARG | -1.22 |
| 4-2 | GLN61>LYS | -1.11 |
| 5-1 | HIS62>ARG | -0.77 |
| 5-2 | HIS62>LYS | -2.03 |
| 6-1 | ASN135>ARG | -0.89 |
| 6-2 | ASN135>HIS | -1.06 |
| 6-3 | ASN135>LYS | -0.91 |
| 7-1 | GLY187>ARG | -1.37 |
| 7-2 | GLY187>LYS | -0.63 |
| 8-1 | MET189>HIS | -2.31 |
| 8-2 | MET189>LYS | -2.08 |
| 9-1 | LEU218>ARG | -2.41 |
| 9-2 | LEU218>LYS | -1.96 |

**Table S6.** The conservation of HemD residues within a distance of 5 Å around HMB molecule analyzed by Hotspot Wizard (The mutable score reflects the conservation of residues, with a lower score indicating higher conservation. Mutable score under 3 was considered highly conserved)

| Residues | Mutable score |
|---|---|
| ARG7 | 1 |
| PRO8 | 1 |
| ILE32 | 4 |
| PHE34 | 4 |
| LEU59 | 4 |
| SER60 | 1 |
| GLN61 | 5 |
| HIS62 | 4 |
| ALA63 | 2 |
| ILE87 | 3 |
| THR91 | 2 |
| SER112 | 2 |
| ARG133 | 3 |
| GLY134 | 2 |
| ASN135 | 5 |
| GLY136 | 4 |
| GLY137 | 2 |
| ARG138 | 1 |
| ILE141 | 3 |
| TYR158 | 1 |
| ARG160 | 1 |
| THR185 | 3 |
| SER186 | 1 |
| GLY187 | 6 |

| | |
|---|---|
| GLU188 | 3 |
| MET189 | 6 |
| ARG217 | 1 |
| LEU218 | 4 |

**Supplementary notes**

**The sequences of genes used in this study**

1. The sequence of *hemD*

ATGAGTATCCTGGTCACCCGCCCGTCTCCCGCTGGAGAAGAGTTAGTGAGC
CGTCTGCGCACACTGGGGCAGGTGGCCTGGCATTTTCCACTGATTGAGTTT
TCTCCGGGTCGACAATTACCACAACTTGCTGATCAACTGGCGGCGCTGGGG
GAGAGCGATCTGTTGTTTGCCCTCTCGCAACACGCGGTTGCTTTTGCCCAA
TCACAGCTGCATCAGCAAGATCGTAAATGGCCCCGACTACCTGATTATTTCG
CCATTGGACGCACCACCGCACTGGCACTACATACCGTAAGCGGACAGAAG
ATTCTCTACCCGCAGGATCGGGAAATCAGCGAAGTCTTGCTACAATTACCT
GAATTACAAAATATTGCGGGCAAACGTGCGCTGATATTACGTGGCAATGGC
GGTCGTGAGCTAATTGGGGATACCCTGACGGCGCGCGGTGCTGAGGTCACT
TTTTGTGAATGTTATCAACGATGCGCAATCCATTACGATGGTGCAGAAGAA
GCGATGCGCTGGCAATCCCGCGAGGTGACGACGGTCGTTGTTACCAGCGG
TGAAATGTTGCAGCAACTCTGGTCGCTGATCCCACAATGGTATCGTGAGCA
CTGGTTACTACACTGTCGACTATTGGTCGTCAGTGAGCGTTTGGCGAAACT
CGCCCGGGAACTGGGCTGGCAAGACATTAAGGTCGCCGATAACGCTGACA
ACGATGCGCTTTTACGGGCATTACAATAA

2. The sequence of *hemE* (*E. coli*)

ATGACCGAACTTAAAAACGATCGTTATCTGCGGGCGCTGCTGCGCCAGCCC
GTTGATGTCACTCCAGTATGGATGATGCGCCAGGCGGGTCGCTATCTACCGG
AATATAAAGCCACGCGCGCCCAGGCGGGCGATTTTATGTCGCTGTGCAAAA
ACGCCGAGCTGGCGTGCGAAGTGACTTTGCAACCGCTGCGTCGCTACCCG
CTGGATGCGGCGATCCTCTTTTCCGATATCCTCACCGTGCCGGACGCGATGG
GGTTAGGGCTCTATTTTGAAGCCGGAGAAGGTCCGCGTTTTACCTCGCCAG
TCACCTGCAAAGCCGACGTCGATAAACTGCCAATTCCGGACCCGGAAGAT
GAGCTGGGTTACGTGATGAACGCGGTGCGTACCATTCGTCGCGAACTGAA
AGGCGAAGTGCCGCTGATTGGTTTTTCCGGCAGCCCGTGGACGCTGGCGA

CCTACATGGTGGAAGGCGGCAGCAGCAAAGCGTTCACCGTGATCAAAAA
ATGATGTATGCCGATCCGCAGGCGCTGCACGCTCTACTCGATAAACTGGCG
AAAAGCGTCACTTTGTATCTGAATGCGCAGATTAAAGCCGGTGCTCAGGCA
GTGATGATTTTCGACACCTGGGGCGGTGTGCTTACCGGGCGCGATTATCAA
CAGTTCTCGCTCTATTACATGCATAAAATTGTTGATGGTTTACTGCGTGAAA
ACGACGGTCGCCGCGTACCGGTCACGCTGTTTACCAAAGGCGGCGGACAG
TGGCTGGAAGCGATGGCAGAAACCGGTTGCGATGCGTTGGGCCTCGACTG
GACAACGGATATCGCCGATGCGCGCCGCCGTGTGGGCAATAAAGTCGCGTT
GCAGGGTAATATGGATCCGTCGATGCTGTACGCTCCGCCTGCCCGCATTGAA
GAAGAAGTAGCGACTATACTTGCAGGTTTCGGTCACGGCGAAGGTCATGTC
TTTAACCTTGGTCACGGCATTCATCAGGATGTGCCGCCAGAACATGCTGGC
GTATTCGTGGAGGCAGTGCATCGACTGTCTGAACAATATCACCGCTAA

3. The sequence of *hemE* (*B. subtilis*)

ATGAGTAAACGAGAAACGTTTAACGAGACGTTTTTAAAAGCTGCGCGGGG
AGAAAAAGCGGATCACACGCCTGTGTGGTATATGAGGCAAGCAGGGCGCT
CACAGCCGGAATACCGCAAGCTGAAGGAAAAGTACGGATTGTTTGAGATC
ACACATCAGCCCGAACTTTGTGCGTATGTCACAAGACTGCCGGTTGAGCAA
TACGGAGTCGATGCTGCAATCCTTTATAAAGATATCATGACGCCGCTGCCGT
CAATCGGTGTGGATGTTGAAATCAAAAACGGGATCGGTCCTGTGATTGATC
AGCCAATCCGGTCTCTGGCGGACATTGAAAAACTCGGCCAGATTGATCCGG
AACAGGACGTGCCGTACGTGCTTGAGACGATTAAACTGCTTGTCAATGAGC
AGCTGAACGTCCCGCTCATCGGTTTCTCAGGTGCGCCTTTTACGCTTGCAA
GCTATATGATCGAAGGCGGCCCGTCGAAAAACTACAATAAAACAAAAGCCT
TCATGTACAGCATGCCAGATGCATGGAATCTGCTGATGTCCAAGCTTGCCG
ACATGATCATCGTGTACGTGAAAGCGCAGATTGAGGCAGGCGCAAAAGCG
ATTCAAATCTTTGATTCGTGGGTCGGCGCATTGAATCAGGCAGATTACAGA
ACATACATCAAACCCGTGATGAACCGGATCTTTTCAGAGCTCGCAAAGGAG
AATGTGCCGCTGATCATGTTTGGCGTTGGCGCAAGCCATCTTGCAGGTGAT

TGGCATGACCTTCCTCTCGATGTTGTCGGGCTTGACTGGAGACTCGGCATT
GATGAAGCCAGATCAAAAGGAATTACTAAAACAGTGCAGGGCAACCTGGA
CCCGTCCATTTTGCTTGCGCCATGGGAAGTCATTGAGCAGAAAACGAAGG
AAATACTTGATCAGGGTATGGAGTCAGACGGCTTCATTTTCAATCTTGGCCA
TGGGGTATTTCCTGATGTCAGTCCCGAGGTTTTGAAAAAACTGACAGCATT
TGTCCATGAATATTCACAAAACAAAAAAATGGGTCAATATTCCTAA

4. The sequence of *hemE* (*C. glutamicum*)

ATGTCTGCTCTTACTATTCCAGCTGCGCGTCGCACGCTAAATAACGCGCCCA
TTATTGATGCCGCTAATGGCAAGACCCCGACTCGCACTCCGGTGTGGTTTAT
GCGCCAGGCGGGTAGGTCGTTGCCTGAGTACAAGAAGGTCCGTGAGGGAA
TCAGCATGTTGGATTCCTGTTTCATGCCGGAGTTGTTGGCGGAGATTACTTT
GCAGCCGGTTCGTCGTCATGATGTGGATGCTGCGATTTTGTTCTCTGACATT
GTGGTGCCGTTGCGTGCTGCGGGGGTTGGTGTGGAAATCGTGGCGGGTCG
TGGACCTGTGTTGGATGCGCCGGTGCGGAGCCGTGAGGATGTGTTGAATCT
TCCTATTTTGGACGGCAACGTTCCGGAGGTGGAGCAGGGTATTGGCATCAT
TTTGGATGAGTTGTCTGATTCTCAGGCGTTGATTGGTTTTGCTGGTGCGCCG
TTTACGTTGGCGAGTTACTTGGTTGAGGGTGGTCCTTCCAAGAATCATGAG
AAAACCAAAGCAATGATGCATGGTGATCCTGAGACGTGGCATGCGTTGATG
GCTCGTTTGGTGCCGACGATTGTGAATTCTTTGAAGTCGCAGATCGACGCG
GGCATCGATGCGATGCAGTTGTTTGATTCGTGGGCTGGGTTCCTCACTGAG
CGTGATTACACCGAGTTCGTGTTGCCGTATTCCACTGAGATTTTGGAGGAA
GTGGGTAAGTACCAGCTGCCTCGTATTCACTTTGGTGTGGGTACTGGTGAG
TTGCTTGGTGCGATGAGCAAGGCTGGCTCAGAGGTCATGGGTGTGGATTGG
CGGGTGCCGTTGGATAAGGCTGCGGAGCGTATTACTGCGGTATCAGGTCCT
AAGGTGTTGCAGGGTAACCTCGATCCTGCGTTGTTGTTTGCGGGTCGCGCA
CCTTTGACTAAGGAAATTGAGCGCATCAAGGCAGAGGCTCAGACTGCTATT
GATGCAGGTCATGCAACGGGCCATATCTTTAACCTTGGTCATGGTGTGCTTC
CTAATACGGTGGCGGAAGATATTACTGAAGCCGTCTCCATCATTCATTCTTA

A

5. The sequence of *hemC*

ATGTTAGACAATGTTTTAAGAATTGCCACACGCCAAAGCCCACTTGCACTC
TGGCAGGCACACTATGTCAAAGACAAGTTGATGGCGAGCCATCCGGGCCT
GGTCGTTGAACTGGTACCGATGGTGACGCGCGGCGATGTGATTCTTGATAC
GCCGCTGGCGAAAGTAGGCGGAAAAGGCTTATTTGTTAAAGAGCTGGAAG
TCGCGCTCCTCGAAAATCGCGCCGATATCGCCGTACATTCAATGAAAGATGT
GCCGGTTGAATTCCCGCAAGGTCTGGGACTGGTCACTATTTGTGAGCGTGA
AGATCCTCGCGATGCCTTTGTGTCCAATAACTATGACAATCTGGATGCGTTA
CCGGCAGGCAGTATCGTCGGGACGTCCAGTTTACGTCGCCAGTGCCAACTG
GCTGAACGCCGCCCGGATCTGATTATCCGCTCCCTGCGAGGCAACGTCGGC
ACTCGCCTGAGTAAACTGGATAACGGCGAATACGATGCCATCATTCTTGCG
GTAGCCGGACTAAAACGTTTAGGTCTGGAGTCCCGCATTCGCGCCGCATTG
CCACCCGAGATTTCTCTTCCGGCGGTAGGACAAGGTGCGGTGGGTATTGAA
TGCCGCCTTGATGATTCTCGCACTCGCGAGCTGCTTGCCGCGCTGAATCAC
CACGAAACTGCACTGCGCGTTACCGCAGAACGCGCCATGAATACCCGTCTC
GAAGGCGGATGTCAGGTGCCAATTGGTAGCTACGCCGAGCTTATTGATGGC
GAAATCTGGCTGCGTGCGTTGGTCGGCGCGCCGGACGGTTCGCAGATTATT
CGCGGTGAACGCCGCGGTGCGCCGCAAGATGCCGAACAAATGGGGATTTC
GCTGGCAGAAGAGCTACTGAATAACGGCGCGCGCGAGATCCTCGCTGAAG
TCTATAACGGAGACGCTCCGGCATAA

6. The sequence of CipA

ATTAATGATATGCACCCCTCACTAATAAAGGACAAGGACATCGTGGATGATG
TGATGCTGCGTAGCTGTAAAATCATCGCTATGAAGGTCATGCCGGATAAGGT
GATGCAGGTTATGGTTACGGTGTTAATGCACGATGGTGTTTGTGAAGAGAT
GTTGCTGAAATGGAATCTGTTGGACAACCGCGGTATGGCAATTTATAAAGT
CCTGATGGAGGCGCTCTGCGAAAAAGGACGTGAAGATCTCCACCGTTG

GCAAAGTAGGTCCGCTGGGCTGCGACTACATTAACTGCGTTGAAATTAGCA
TG

7. The sequence of CipB

ATGATTATAAAGAAAGATATACTATTAAATGAAGAGCTGATTGTGGATGACG
ATCTGAAGGTGGGTAAAGTCGAAAAAGTGAACATTGATATCCTGTCGCCGT
CTAGCGTTATCGTGAGCCTGAATATTCTGGGTGTTGTGGACGACTTCCACTT
GTTGTTGGTTGACGACAAGGACAAGGATAAGATCGTGTTGTTATACCTGTC
CCTGCTCCGTGTTCTGCATGAGAAGCTGGATGTTAAGGTCAAAGTTGCGAA
AAGCAAACTTACCAAAATCAAATATATTGTGGGCGTAGAGATC

8. The sequence of SAP

GCTAATGCGAATGCCAGAGCAAGGGCTAATGCGAACGCTCGCGCACGT

9. The sequence of spycatcher

GCTATGGTTGATACCCTGTCCGGCCTGAGCTCTGAACAGGGCCAAAGCGGT
GACATGACCATTGAAGAGGATAGCGCGACGCATATCAAATTCAGCAAGCGC
GACGAGGACGGCAAAGAGTTGGCGGGTGCTACGATGGAACTCCGTGACTC
GAGCGGCAAGACCATCTCCACCTGGATTTCTGATGGTCAAGTGAAAGATTT
TTATCTGTACCCGGGTAAGTACACCTTCGTGGAGACAGCGGCACCGGATGG
CTATGAAGTTGCAACTGCGATTACCTTTACCGTGAACGAGCAGGGTCAGGT
CACCGTTAATGGTAAGGCGACCAAAGGTGACGCCCACATC

10. The sequence of spytag

GCTCACATAGTAATGGTTGATGCATATAAACCGACCAAG

11. The sequence of snoopcatcher

GCGAGCAAACCGCTGCGCGGCGCCGTTTTTCCTTACAAAAGCAGCATCCG
GATTACCCGGACATTTACGGTGCGATCGACCAAAACGGCACCTACCAGAAC

GTTCGTACGGGCGAAGACGGCAAGCTGACCTTTAAAAACTTGTCGGACGG
TAAGTATCGTCTGTTCGAGAACAGCGAACCGGCGGGTTATAAACCGGTTCA
GAATAAACCGATCGTGGCTTTCCAGATCGTCAACGGCGAAGTGCGTGATGT
TACCAGCATTGTGCCACAAGATATTCCGGCAACTTACGAGTTCACCAATGGT
AAGCACTATATCACCAATGAGCCGATTCCGCCTAAG

12. The sequence of snooptag
GCTAGTAAACTAGGAGATATTGAATTTATAAAGGTGAACAAA

13. The sequence of RIAD
CTGGAACAGTATGCAAATCAGCTGGCAGATCAGATTATCAAAGAAGCAACC
GAAGGTTGC

14. The sequence of RIDD
AGCCTGCGTGAATGTGAACTGTATGTTCAGAAACATAATATTCAGGCCCTGC
TGAAAGATAGCATTGTTCAGCTGTGTACCGCACGTCCGGAACGTCCGATGG
CATTTCTGCGCGAATATTTTGAACGTCTGGAAAAAGAAGAAGCCAAA

15. The sequence of HemC-L1-HemD
ATGTTAGACAATGTTTTAAGAATTGCCACACGCCAAAGCCCACTTGCACTC
TGGCAGGCACACTATGTCAAAGACAAGTTGATGGCGAGCCATCCGGGCCT
GGTCGTTGAACTGGTACCGATGGTGACGCGCGGCGATGTGATTCTTGATAC
GCCGCTGGCGAAAGTAGGCGGAAAAGGCTTATTTGTTAAAGAGCTGGAAG
TCGCGCTCCTCGAAAATCGCGCCGATATCGCCGTACATTCAATGAAAGATGT
GCCGGTTGAATTCCCGCAAGGTCTGGGACTGGTCACTATTTGTGAGCGTGA
AGATCCTCGCGATGCCTTTGTGTCCAATAACTATGACAATCTGGATGCGTTA
CCGGCAGGCAGTATCGTCGGGACGTCCAGTTTACGTCGCCAGTGCCAACTG
GCTGAACGCCGCCCGGATCTGATTATCCGCTCCCTGCGAGGCAACGTCGGC
ACTCGCCTGAGTAAACTGGATAACGGCGAATACGATGCCATCATTCTTGCG

GTAGCCGGACTAAAACGTTTAGGTCTGGAGTCCCGCATTCGCGCCGCATTG
CCACCCGAGATTTCTCTTCCGGCGGTAGGACAAGGTGCGGTGGGTATTGAA
TGCCGCCTTGATGATTCTCGCACTCGCGAGCTGCTTGCCGCGCTGAATCAC
CACGAAACTGCACTGCGCGTTACCGCAGAACGCGCCATGAATACCCGTCTC
GAAGGCGGATGTCAGGTGCCAATTGGTAGCTACGCCGAGCTTATTGATGGC
GAAATCTGGCTGCGTGCGTTGGTCGGCGCGCCGGACGGTTCGCAGATTATT
CGCGGTGAACGCCGCGGTGCGCCGCAAGATGCCGAACAAATGGGGATTTC
GCTGGCAGAAGAGCTACTGAATAACGGCGCGCGCGAGATCCTCGCTGAAG
TCTATAACGGAGACGCTCCGGCAGGTGGTGGTAGCATGAGTATCCTGGTCA
CCCGCCCGTCTCCCGCTGGAGAAGAGTTAGTGAGCCGTCTGCGCACACTG
GGGCAGGTGGCCTGGCATTTTCCACTGATTGAGTTTTCTCCGGGTCGACAA
TTACCACAACTTGCTGATCAACTGGCGGCGCTGGGGGAGAGCGATCTGTTG
TTTGCCCTCTCGCAACACGCGGTTGCTTTTGCCCAATCACAGCTGCATCAG
CAAGATCGTAAATGGCCCCGACTACCTGATTATTTCGCCATTGGACGCACCA
CCGCACTGGCACTACATACCGTAAGCGGACAGAAGATTCTCTACCCGCAGG
ATCGGGAAATCAGCGAAGTCTTGCTACAATTACCTGAATTACAAAATATTGC
GGGCAAACGTGCGCTGATATTACGTGGCAATGGCGGTCGTGAGCTAATTGG
GGATACCCTGACGGCGCGCGGTGCTGAGGTCACTTTTTGTGAATGTTATCA
ACGATGCGCAATCCATTACGATGGTGCAGAAGAAGCGATGCGCTGGCAATC
CCGCGAGGTGACGACGGTCGTTGTTACCAGCGGTGAAATGTTGCAGCAAC
TCTGGTCGCTGATCCCACAATGGTATCGTGAGCACTGGTTACTACACTGTCG
ACTATTGGTCGTCAGTGAGCGTTTGGCGAAACTCGCCCGGGAACTGGGCT
GGCAAGACATTAAGGTCGCCGATAACGCTGACAACGATGCGCTTTTACGGG
CATTACAATAA

16. The sequence of HemC-L2-HemD

ATGTTAGACAATGTTTTAAGAATTGCCACACGCCAAAGCCCACTTGCACTC
TGGCAGGCACACTATGTCAAAGACAAGTTGATGGCGAGCCATCCGGGCCT
GGTCGTTGAACTGGTACCGATGGTGACGCGCGGCGATGTGATTCTTGATAC

GCCGCTGGCGAAAGTAGGCGGAAAAGGCTTATTTGTTAAAGAGCTGGAAG
TCGCGCTCCTCGAAAATCGCGCCGATATCGCCGTACATTCAATGAAAGATGT
GCCGGTTGAATTCCCGCAAGGTCTGGGACTGGTCACTATTTGTGAGCGTGA
AGATCCTCGCGATGCCTTTGTGTCCAATAACTATGACAATCTGGATGCGTTA
CCGGCAGGCAGTATCGTCGGGACGTCCAGTTTACGTCGCCAGTGCCAACTG
GCTGAACGCCGCCCGGATCTGATTATCCGCTCCCTGCGAGGCAACGTCGGC
ACTCGCCTGAGTAAACTGGATAACGGCGAATACGATGCCATCATTCTTGCG
GTAGCCGGACTAAAACGTTTAGGTCTGGAGTCCCGCATTCGCGCCGCATTG
CCACCCGAGATTTCTCTTCCGGCGGTAGGACAAGGTGCGGTGGGTATTGAA
TGCCGCCTTGATGATTCTCGCACTCGCGAGCTGCTTGCCGCGCTGAATCAC
CACGAAACTGCACTGCGCGTTACCGCAGAACGCGCCATGAATACCCGTCTC
GAAGGCGGATGTCAGGTGCCAATTGGTAGCTACGCCGAGCTTATTGATGGC
GAAATCTGGCTGCGTGCGTTGGTCGGCGCGCCGGACGGTTCGCAGATTATT
CGCGGTGAACGCCGCGGTGCGCCGCAAGATGCCGAACAAATGGGGATTTC
GCTGGCAGAAGAGCTACTGAATAACGGCGCGCGAGATCCTCGCTGAAG
TCTATAACGGAGACGCTCCGGCAGGTGGTAGCGGTGGTGGTAGCGGTATGA
GTATCCTGGTCACCCGCCCGTCTCCCGCTGGAGAAGAGTTAGTGAGCCGTC
TGCGCACACTGGGGCAGGTGGCCTGGCATTTTCCACTGATTGAGTTTTCTC
CGGGTCGACAATTACCACAACTTGCTGATCAACTGGCGGCGCTGGGGGAG
AGCGATCTGTTGTTTGCCCTCTCGCAACACGCGGTTGCTTTTGCCCAATCA
CAGCTGCATCAGCAAGATCGTAAATGGCCCCGACTACCTGATTATTTCGCCA
TTGGACGCACCACCGCACTGGCACTACATACCGTAAGCGGACAGAAGATTC
TCTACCCGCAGGATCGGGAAATCAGCGAAGTCTTGCTACAATTACCTGAAT
TACAAAATATTGCGGGCAAACGTGCGCTGATATTACGTGGCAATGGCGGTC
GTGAGCTAATTGGGGATACCCTGACGGCGCGCGGTGCTGAGGTCACTTTTT
GTGAATGTTATCAACGATGCGCAATCCATTACGATGGTGCAGAAGAAGCGA
TGCGCTGGCAATCCCGCGAGGTGACGACGGTCGTTGTTACCAGCGGTGAA
ATGTTGCAGCAACTCTGGTCGCTGATCCCACAATGGTATCGTGAGCACTGG
TTACTACACTGTCGACTATTGGTCGTCAGTGAGCGTTTGGCGAAACTCGCC

CGGGAACTGGGCTGGCAAGACATTAAGGTCGCCGATAACGCTGACAACGA
TGCGCTTTTACGGGCATTACAATAA

17. The sequence of HemC-L1-HemD

ATGTTAGACAATGTTTTAAGAATTGCCACACGCCAAAGCCCACTTGCACTC
TGGCAGGCACACTATGTCAAAGACAAGTTGATGGCGAGCCATCCGGGCCT
GGTCGTTGAACTGGTACCGATGGTGACGCGCGGCGATGTGATTCTTGATAC
GCCGCTGGCGAAAGTAGGCGGAAAAGGCTTATTTGTTAAAGAGCTGGAAG
TCGCGCTCCTCGAAAATCGCGCCGATATCGCCGTACATTCAATGAAAGATGT
GCCGGTTGAATTCCCGCAAGGTCTGGGACTGGTCACTATTTGTGAGCGTGA
AGATCCTCGCGATGCCTTTGTGTCCAATAACTATGACAATCTGGATGCGTTA
CCGGCAGGCAGTATCGTCGGGACGTCCAGTTTACGTCGCCAGTGCCAACTG
GCTGAACGCCGCCCGGATCTGATTATCCGCTCCCTGCGAGGCAACGTCGGC
ACTCGCCTGAGTAAACTGGATAACGGCGAATACGATGCCATCATTCTTGCG
GTAGCCGGACTAAAACGTTTAGGTCTGGAGTCCCGCATTCGCGCCGCATTG
CCACCCGAGATTTCTCTTCCGGCGGTAGGACAAGGTGCGGTGGGTATTGAA
TGCCGCCTTGATGATTCTCGCACTCGCGAGCTGCTTGCCGCGCTGAATCAC
CACGAAACTGCACTGCGCGTTACCGCAGAACGCGCCATGAATACCCGTCTC
GAAGGCGGATGTCAGGTGCCAATTGGTAGCTACGCCGAGCTTATTGATGGC
GAAATCTGGCTGCGTGCGTTGGTCGGCGCGCCGGACGGTTCGCAGATTATT
CGCGGTGAACGCCGCGGTGCGCCGCAAGATGCCGAACAAATGGGGATTTC
GCTGGCAGAAGAGCTACTGAATAACGGCGCGCGCGAGATCCTCGCTGAAG
TCTATAACGGAGACGCTCCGGCAGGTGGTGGTGGTAGCGGTGGTGGTGGT
AGCGGTGGTGGTGGTAGCATGAGTATCCTGGTCACCCGCCCGTCTCCCGCT
GGAGAAGAGTTAGTGAGCCGTCTGCGCACACTGGGGCAGGTGGCCTGGCA
TTTTCCACTGATTGAGTTTTCTCCGGGTCGACAATTACCACAACTTGCTGAT
CAACTGGCGGCGCTGGGGGAGAGCGATCTGTTGTTTGCCCTCTCGCAACA
CGCGGTTGCTTTTGCCCAATCACAGCTGCATCAGCAAGATCGTAAATGGCC
CCGACTACCTGATTATTTCGCCATTGGACGCACCACCGCACTGGCACTACAT

ACCGTAAGCGGACAGAAGATTCTCTACCCGCAGGATCGGGAAATCAGCGA
AGTCTTGCTACAATTACCTGAATTACAAAATATTGCGGGCAAACGTGCGCT
GATATTACGTGGCAATGGCGGTCGTGAGCTAATTGGGGATACCCTGACGGC
GCGCGGTGCTGAGGTCACTTTTTGTGAATGTTATCAACGATGCGCAATCCAT
TACGATGGTGCAGAAGAAGCGATGCGCTGGCAATCCCGCGAGGTGACGAC
GGTCGTTGTTACCAGCGGTGAAATGTTGCAGCAACTCTGGTCGCTGATCCC
ACAATGGTATCGTGAGCACTGGTTACTACACTGTCGACTATTGGTCGTCAGT
GAGCGTTTGGCGAAACTCGCCCGGGAACTGGGCTGGCAAGACATTAAGGT
CGCCGATAACGCTGACAACGATGCGCTTTTACGGGCATTACAATAA

18. The sequence of *hemA*

ATGACCCTTTTAGCACTCGGTATCAACCATAAAACGGCACCTGTATCGCTGC
GAGAACGTGTATCGTTTTCGCCGGATAAGCTCGATCAGGCGCTTGACAGCC
TGCTTGCGCAGCCGATGGTGCAGGGCGGCGTGGTGCTGTCGACGTGCAAC
CGCACGGAACTTTATCTTAGCGTTGAAGAGCAGGATAACCTGCAAGAGGC
GTTAATCCGCTGGCTTTGCGATTATCACAATCTTAATGAAGAAGATCTGCGT
AAAAGCCTCTACTGGCATCAGGATAACGACGCGGTTAGCCATTTAATGCGT
GTTGCCAGCGGCCTGGATTCATTGGTTCTTGGGGAGCCGCAGATCCTCGGT
CAGGTTAAAAAAGCGTTTGCCGATTCGCAAAAAGGCCATATGAAGGCCAG
CGAACTGGAACGCATGTTCCAGAAATCTTTCTCTGTAGCGAAACGCGTTCG
CACTGAAACAGATATCGGTGCCAGCGCTGTGTCTGTCGCTTTTGCGGCTTG
TACGCTGGCGCGGCAGATCTTTGAATCGCTCTCTACGGTCACAGTGTTGCT
GGTAGGCGCGGGCGAAACCATCGAGCTGGTAGCGCGTCATCTGCGCGAAC
ATAAAGTACAGAAGATGATTATCGCCAACCGCACTCGCGAACGTGCCCAAA
TACTGGCAGATGAAGTTGGCGCGGAAGTGATTGCCCTGAGTGAGATCGAC
GAACGTCTGCGCGAAGCCGATATCATCATCAGTTCCACCGCCAGCCCGTTA
CCGATTATCGGGAAAGGCATGGTGGAGCGCGCATTAAAAAGCCGTCGCAA
CCAACCAATGCTGTTGGTGGATATTGCCGTTCCGCGCGATGTTGAGCCGGA
AGTTGGCAAACTGGCGAATGCTTATCTTTATAGCGTGGACGATCTGCAAAG

CATCATTTCGCACAACCTGGCGCAGCGTAAAGCCGCAGCGGTTGAGGCGG
AAACTATTGTCGCTCAGGAAACCAGCGAATTTATGGCGTGGCTGCGAGCAC
AAAGCGCCAGCGAAACCATTCGCGAGTATCGCAGCCAGGCAGAGCAAGTT
CGCGATGAGTTAACCGCCAAAGCGTTAGCGGCCCTTGAGCAGGGCGGCGA
CGCGCAAGCCATTATGCAGGATCTGGCATGGAAACTGACTAACCGCTTGAT
CCATGCGCCAACGAAATCACTTCAACAGGCCGCCCGTGACGGGGATAACG
AACGCCTGAATATTCTGCGCGACAGCCTCGGGCTGGAGTAG

19. The sequence of *hemL*

ATGAGTAAGTCTGAAAATCTTTACAGCGCAGCGCGCGAGCTGATCCCTGGC
GGTGTGAACTCCCCTGTTCGCGCCTTTACTGGCGTGGGCGGCACTCCACTG
TTTATCGAAAAAGCGGACGGCGCTTATCTGTACGATGTTGATGGCAAAGCC
TATATCGATTATGTCGGTTCCTGGGGGCCGATGGTGCTGGGCCATAACCATC
CGGCAATCCGCAATGCCGTGATTGAAGCCGCCGAGCGTGGTTTAAGCTTTG
GTGCACCAACCGAAATGGAAGTGAAAATGGCGCAACTGGTGACTGAACTG
GTCCCGACCATGGATATGGTGCGCATGGTGAACTCCGGCACCGAGGCGACG
ATGAGCGCCATCCGCCTGGCCCGTGGTTTTACCGGTCGCGACAAAATTATTA
AATTTGAAGGTTGTTACCACGGTCACGCTGACTGCCTGCTGGTGAAAGCCG
GTTCTGGCGCACTCACGTTAGGCCAGCCAAACTCGCCGGGCGTTCCGGCA
GATTTCGCCAAACATACCTTAACCTGTACTTATAACGATCTGGCTTCTGTAC
GCGCCGCGTTTGAGCAATACCCGCAAGAGATTGCCTGTATTATCGTCGAGC
CGGTGGCAGGCAATATGAACTGCGTTCCACCGCTGCCAGAGTTCCTGCCAG
GTCTGCGTGCGCTGTGCGACGAATTTGGCGCATTGCTGATCATCGATGAAG
TAATGACCGGCTTCCGCGTGGCACTGGCTGGCGCACAGGATTATTACGGTG
TGGAACCGGATCTCACCTGCCTGGGCAAAATCATCGGCGGTGGAATGCCG
GTAGGCGCATTCGGTGGTCGTCGTGATGTAATGGATGCGCTGGCCCCGACG
GGTCCGGTCTATCAGGCGGGTACGCTTTCCGGTAACCCAATTGCGATGGCA
GCGGGTTTCGCCTGTCTGAATGAAGTCGCGCAGCCGGGCGTTCACGAAAC
GTTGGATGAGCTGACATCACGTCTGGCAGAAGGTCTGCTGGAAGCGGCAG

AAGAAGCCGGAATTCCGCTGGTCGTTAACCACGTTGGCGGCATGTTCGGTA
TTTTCTTTACCGACGCCGAGTCCGTGACGTGCTATCAGGATGTGATGGCCTG
TGACGTGGAACGCTTTAAGCGTTTCTTCCATATGATGCTGGACGAAGGTGT
TTACCTGGCACCGTCAGCGTTTGAAGCGGGCTTTATGTCCGTGGCGCACAG
CATGGAAGATATCAATAACACCATCGATGCTGCACGTCGGGTGTTTGCGAA
GTTGTGA
